# High expression of SLC20A1 is less effective for endocrine therapy and predicts late recurrence in ER-positive breast cancer

**DOI:** 10.1371/journal.pone.0268799

**Published:** 2022-05-23

**Authors:** Chotaro Onaga, Shoma Tamori, Izumi Matsuoka, Ayaka Ozaki, Hitomi Motomura, Yuka Nagashima, Tsugumichi Sato, Keiko Sato, Yuyun Xiong, Kazunori Sasaki, Shigeo Ohno, Kazunori Akimoto

**Affiliations:** 1 Department of Medicinal and Life Sciences, Faculty of Pharmaceutical Sciences, Tokyo University of Science, Chiba, Japan; 2 Department of Pharmacy, Faculty of Pharmaceutical Sciences, Tokyo University of Science, Chiba, Japan; 3 Department of Information Sciences, Faculty of Sciences and Technology, Tokyo University of Science, Chiba, Japan; 4 Laboratory of Cancer Biology, Institute for Diseases of Old Age, Juntendo University School of Medicine, Tokyo, Japan; University of Wisconsin-Madison, UNITED STATES

## Abstract

Estrogen receptor-positive (ER+) breast cancer intrinsically confers satisfactory clinical outcomes in response to endocrine therapy. However, a significant proportion of patients with ER+ breast cancer do not respond well to this treatment. Therefore, to evaluate the effects of endocrine therapy, there is a need for identification of novel markers that can be used at the time of diagnosis for predicting clinical outcomes, especially for early-stage and late recurrence. Solute carrier family 20 member 1 (SLC20A1) is a sodium/inorganic phosphate symporter that has been proposed to be a viable prognostic marker for the luminal A and luminal B types of ER+ breast cancer. In the present study, we examined the possible association of SLC20A1 expression with tumor staging, endocrine therapy and chemotherapy in the luminal A and luminal B subtypes of breast cancer. In addition, we analyzed the relationship between SLC20A1 expression and late recurrence in patients with luminal A and luminal B breast cancer following endocrine therapy. We showed that patients with higher levels of *SLC20A1* expression (*SLC20A1*^high^) exhibited poorer clinical outcomes in those with tumor stage I luminal A breast cancer. In addition, this *SLC20A1*^high^ subgroup of patients exhibited less responses to endocrine therapy, specifically in those with the luminal A and luminal B subtypes of breast cancer. However, patients with *SLC20A1*^high^ showed good clinical outcomes following chemotherapy. Patients tested to be in the *SLC20A1*^high^ group at the time of diagnosis also showed a higher incidence of recurrence compared with those with lower expression levels of *SLC20A1*, at >15 years for luminal A breast cancer and at 10–15 years for luminal B breast cancer. Therefore, we conclude that *SLC20A1*^high^ can be used as a prognostic biomarker for predicting the efficacy of endocrine therapy and late recurrence for ER+ breast cancer.

## Introduction

Breast cancer is the most common malignancy among women and the leading cause of cancer-associated mortality in women worldwide [1]. In general, when the cancer is detected early, patients will exhibit longer survival times with less extensive treatment regimens and minimal risk of cancer progression. Breast cancer is one of the most stratified types of cancer, where the treatment methodology is typically designed according to the subtype and tumor stage [2–4]. This stratification has led to improvements in the clinical outcome [2–4]. However, there remains a substantial proportion of patients who do not respond well to treatment. Furthermore, early prediction of patient prognosis provides an opportunity to maximize the range of treatment options available at the earliest possible tumor stage, which can confer significant benefits on the quality of life. Therefore, identification of novel biomarkers that can accurately predict the prognosis of patients with early stage tumors, and in turn optimize the treatment strategy remains in high demand.

Estrogen receptor-positive (ER+) breast cancer is a major breast cancer subtype that accounts for 70–80% of all types of breast cancers [3]. Breast cancer is stratified into ≥ six subtypes in accordance with their gene expression profiles (PAM 50), with the main subtypes being normal-like, luminal A, luminal B, human epidermal growth factor receptor 2 (HER2)-enriched, claudin-low and basal-like [5–9]. In particular, the luminal A and luminal B subtypes fall under the ER+ subtype of breast cancer [10, 11]. Patients with luminal A and luminal B breast cancer subtypes are known to exhibit superior prognosis compared with that in the other subtypes [7–12]. However, 25–50% of the patients with these two subtypes become less responsive to endocrine therapy due to the heterogenous phenotypes of tumor cells and development of resistance to therapy [12, 13]. In addition, another important obstacle blocking the effective treatment of patients with ER+ breast cancer is late recurrence. A small number of patients relapse after >5 years of endocrine therapy [14–21]. In one study, 15-year distant relapse rates are 27.8% for luminal A and 42.9% luminal B breast cancer [17]. It has been previously reported that tumor size and lymph node metastasis are associated with late recurrence [15–21]. However, unlike early recurrence, Ki-67 and p53 expression are not likely to be associated with late recurrence [20, 21]. In fact, there is currently a lack of accurate parameters that can be applied for the prediction of late recurrence. Although dormant micro-metastatic cells have been proposed to be one of the mechanism underlying late recurrence, this hypothesis remains in its infancy [22, 23]. Therefore, identification of a biomarker for clinically predicting late recurrence in patients with ER+ breast cancer after medical treatment is required. In this field, analysis of gene expression profiles in breast cancer and evaluation of their corresponding clinical outcomes have been demonstrated to be beneficial for the systemic stratification of breast cancers [24, 25]. This allowed the degree of tumor heterogeneity among patients to be more accurately reflected [24, 25]. Therefore, biomarkers for predicting the outcome of endocrine therapy and late recurrence in patients with luminal A and luminal B breast cancer are in urgent demand.

Solute carrier family 20 member 1 (SLC20A1) is a sodium/inorganic phosphate (Pi) symporter [26, 27]. The expression of SLC20A1 is high in ER+ breast cancer and has been previously found to associated with poor prognosis [24, 25]. In addition, apart from the ER+ luminal A and luminal B subtypes, patients with higher levels of *SLC20A1* expression (*SLC20A1*^high^) in the claudin-low and basal-like subtypes have been reported to show inferior clinical outcomes. Radiation therapy against *SLC20A1*^high^ claudin-low and basal-like subtypes of breast cancer tumors has been demonstrated to be insufficient [25]. In addition, inhibiting SLC20A1 has been shown to delay cell cycle progression and impair mitosis and cytokinesis and cell proliferation in cancer cells [28]. SLC20A1 knockdown using small interfering RNA (siRNA) also reduced the viability of the luminal A subtype MCF7 cell line [25]. Although, these previous findings suggest the potential prognostic value of using *SLC20A1* expression for predicting late recurrence in patients with ER+ at early tumor stages, its relationship with tumor stage or endocrine therapy outcomes, and late recurrence remain to be clarified.

To assess the potential prognostic implications of *SLC20A1* expression in detail, we evaluated its association with tumor stage and endocrine therapy outcomes in patients with luminal A and luminal B breast cancers in the present study. Additionally, we analyzed the possible relationship between late recurrence and *SLC20A1* expression in patients with luminal A and luminal B breast cancer after endocrine therapy.

## Materials and methods

### Molecular Taxonomy of the Breast Cancer International Consortium dataset

The Molecular Taxonomy of Breast Cancer International Consortium (METABRIC) dataset [29, 30] was downloaded from the cBioportal (http://cbioportal.org) [31, 32] on July 29, 2020. The clinicopathological data from these patients were summarized S1 Table and previously [25, 33, 34]. The average values of the ages at the time of diagnosis in the entire cohort, and in the Luminal A and Luminal B subtypes were as follows [all, 61.09 years (21.93–96.29); luminal A, 62.78 years (26.36–90.23); luminal B, 65.21 years (28.62–92.14)]. This METABRIC dataset contains mRNA expression profile data (n = 1904). We defined the optimal cut-off thresholds using Youden’s index to assign the patients into the *SLC20A1*^high^ and low-expression (*SLC20A1*^low^) groups through receiver operating characteristic (ROC) analysis. ROC was performed by SLC20A1 expression gene and disease-specific survival (DSS) or relapse free status (RFS) for each group divided, and calculated Youden’s index (S2 Table). Patient data of ‘living’ and ‘Died of Disease’ of patient’s vital status were used as DSS and relapse free status were used as RFS.

### Analysis of patient prognosis using the Kaplan-Meier method

Survival curves based on DSS and RFS were plotted using the Kaplan-Meier method. The curves were compared between the *SLC20A1*^high^ and *SLC20A1*^low^ groups using the log-rank (Cochran-Mantel-Haenszel) test. Kaplan-Meier survival curves were produced using BellCurve for Excel version 3.00 (Social Survey Research Information Co., Ltd.).

### Analysis of patient prognosis using the multivariate Cox regression method

Multivariate Cox regression analysis was performed to evaluate the influence of high and low *SLC20A1* gene expression on patient outcome and to estimate the *SLC20A1*^high^ group’s adjusted hazard ratios (HRs) to *SLC20A1*^low^ group for DSS or RFS. The ages at the time of diagnosis were adjusted as a confounding factor to remove the effect of age. We set the level of significance to be at 5% (two-sided). Multivariate Cox regression analyses were carried out using BellCurve for Excel version 3.00 (Social Survey Research Information Co., Ltd.).

### Analysis of the recurrence incidence rate

The recurrence incidence rate was calculated using the number of recurrences divided by the observation term of the patients with luminal A and luminal B breast cancer after endocrine therapy. The observation term was divided every 5 years, where the number of recurrences was then counted during that term. The observation term of the patients was the total observation time (year) during that period. The *p*-value was calculated from the statistic based on normal distribution and corrected using the Holm method. Incidence rate ratio was calculated as the ratio of the recurrence incidence rate of *SLC20A1*^high^ group to that in the *SLC20A1*^low^ group.

## Results

### *SLC20A1*^high^ confers poorer clinical outcomes for patients with early-stage breast cancer according to Kaplan-Meier analysis and multivariate Cox regression analyses

**Fig 1:**
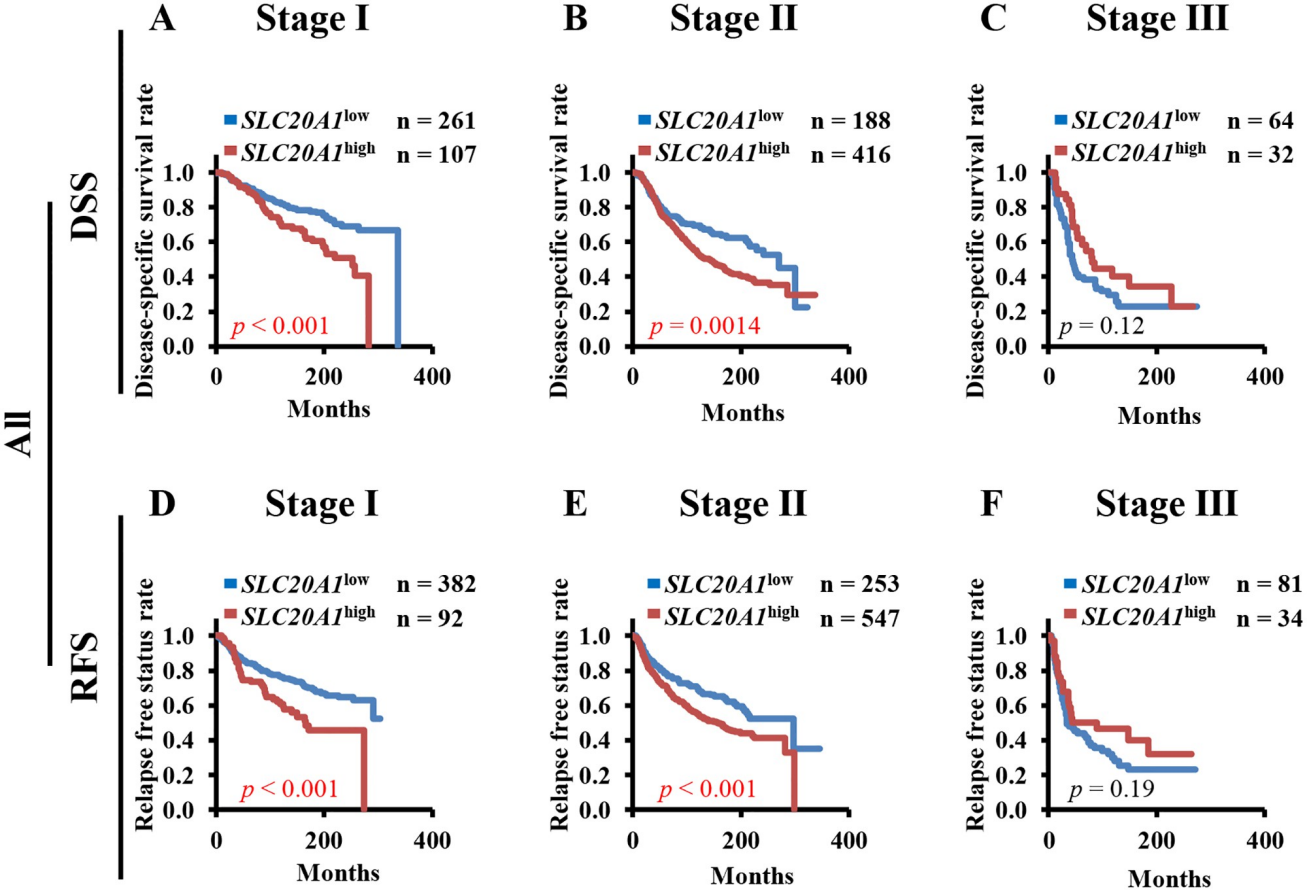
Patients with high solute carrier family 20 member 1 (SLC20A1) expression (*SLC20A1*^high^) have poorer clinical outcomes at early tumor stages. (A-F) Kaplan-Meier analyses comparing the disease-specific survival (DSS) and relapse-free status (RFS) in all patients with breast cancer between those in the *SLC20A1*^high^ and low SLC20A1 expression (*SLC20A1*^low^) groups. (A) DSS in tumor stage I. (B) DSS in tumor stage II. (C) DSS in tumor stage III. (D) RFS in tumor stage I. (E) RFS in tumor stage II. (F) RFS in tumor stage III.

It has been previously shown that the levels of SLC20A1 expression in ER+ breast cancer tissues are higher compared with those in normal tissues, where high *SLC20A1* expression is associated with poorer clinical outcomes [24, 25]. However, the TCGA dataset used in the previous analysis had a small number of patients (n = 526) and did not include endocrine therapy data. And the possible association between SLC20A1 expression at tumor stages and clinical outcomes in patients with breast cancer remains poorly defined. In the present study, therefore, to assess the role of *SLC20A1* expression in ER+ breast cancer subtypes in detail, we analyzed a METABRIC dataset that included the gene expression data from 1904 breast cancer patients. We first compared the levels of *SLC20A1* expression in tissues among the various tumor stages. Box-plot analysis revealed no statistical difference in *SLC20A1* expression among the tumor stages (S1A Fig). Statistical difference in *SLC20A1* expression among the tumor stages in the ER+, luminal A and luminal B subtypes also could not be found (S1B and S1C Fig). We next performed Kaplan-Meier analysis to compare DSS and RFS between patients in the *SLC20A1*^high^ and *SLC20A1*^low^ groups at tumor stages I, II and III. Patients in the *SLC20A1*^high^ group showed poorer clinical outcomes at tumor stage I (DSS, *p*<0.001; RFS, *p*<0.001) (Fig 1A and 1D) and stage II (DSS, *p* = 0.0014; RFS, *p*<0.001) (Fig 1B and 1E). At tumor stage III, patients with *SLC20A1*^high^ did not show significance in their clinical outcomes (DSS, *p* = 0.12; RFS, *p* = 0.19) (Fig 1C and 1F). To verify the results from the Kaplan-Meier analysis, multivariate Cox regression analyses were then performed between the *SLC20A1*^high^ and *SLC20A1*^low^ groups for DSS and RFS with age as a confounding factor. Patients with *SLC20A1*^high^ at tumor stages I and II exhibited poorer clinical outcomes (DSS: stage I, HR = 1.92, 95% CI = 1.30–2.82; stage II, HR = 1.55, 95% CI = 1.18–2.03; RFS: stage I, HR = 1.91, 95% CI = 1.34–2.74; stage II, HR = 1.52, 95% CI = 1.20–1.95); however, this was not the case with patients at tumor stage III (DSS: HR = 0.65, 95% CI = 0.39–1.11; RFS: HR = 0.70, 95% CI = 0.42–1.18) (Table 1). These results strongly suggest that high *SLC20A1* expression may be used as a prognostic biomarker for poor outcomes of patients with early-stage breast cancer.

**Table 1:** Multivariate Cox regression analyses of disease-specific survival (DSS) and relapse-free status (RFS) in tumor stages I, II or III.

Staging	DSS		
	Hazard ratio^a^	95% confidence interval	*P*-value
**Stage I**	**1.92**	**1.30–2.82**	**<0.001**
**Stage II**	**1.55**	**1.18–2.03**	**0.0016**
Stage III	0.65	0.39–1.11	0.11
Staging	RFS		
	Hazard ratio^a^	95% confidence interval	*P*-value
**Stage I**	**1.91**	**1.34–2.74**	**<0.001**
**Stage II**	**1.52**	**1.20–1.95**	**<0.001**
Stage III	0.70	0.42–1.18	0.18

^a^*SLC20A1*^high^ group’s hazard ratio to *SLC20A1*^low^ group adjusted using age as a confounding factor as estimated using Cox proportional hazard model. Significant differences are shown in bold.

### Among patients with early-stage luminal A breast cancer *SLC20A1*^high^ confers poorer clinical outcomes according to Kaplan-Meier analysis and multivariate Cox regression analysis

**Fig 2:**
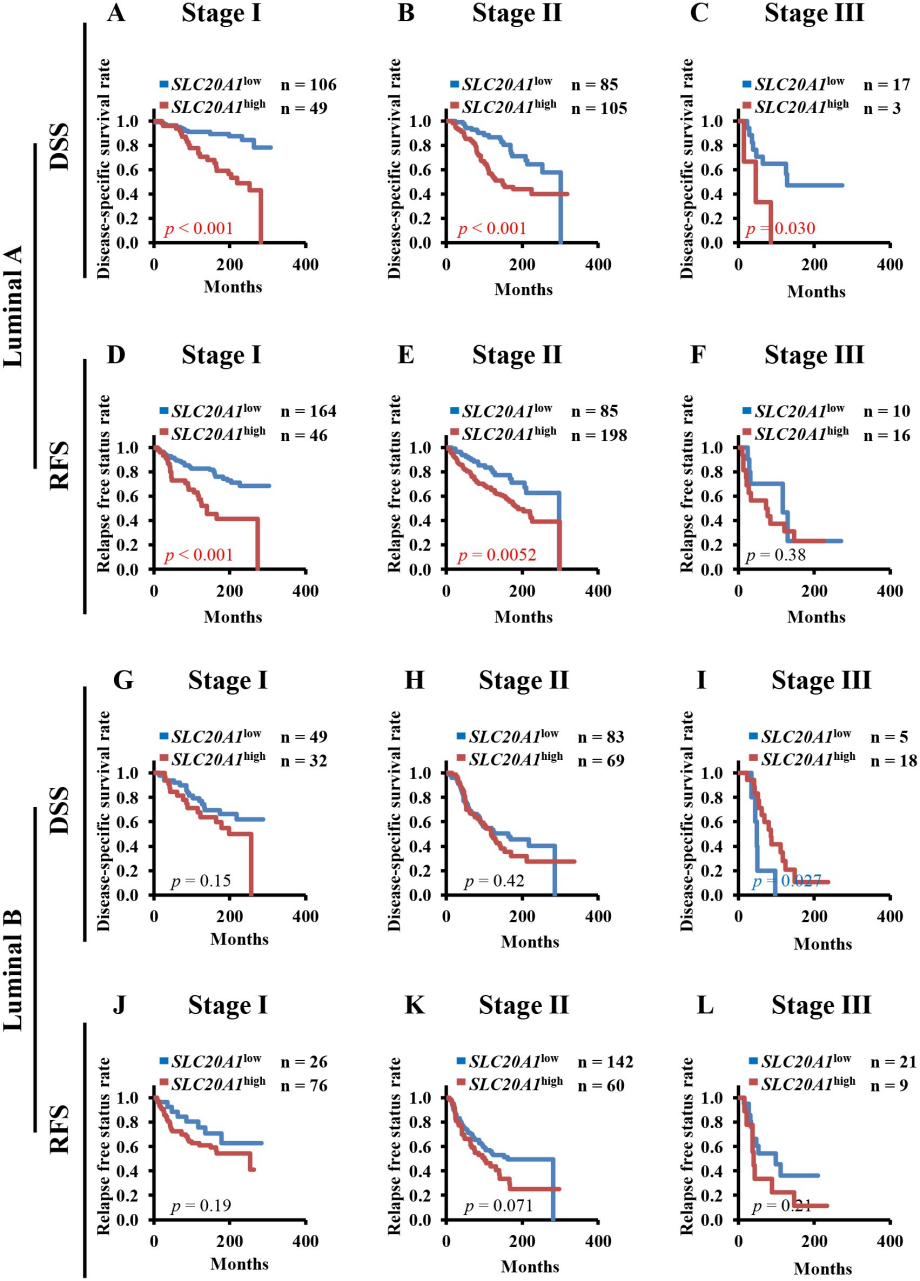
Patients with high solute carrier family 20 member 1 (SLC20A1) expression (*SLC20A1*^high^) have poorer outcomes from early-stage luminal A breast cancer. (A-F) Kaplan-Meier analyses comparing disease-specific survival (DSS) and relapse-free status (RFS) in patients with luminal A breast cancer between the *SLC20A1*^high^ and *SLC20A1*^low^ groups. (A) DSS in tumor stage I. (B) DSS in tumor stage II. (C) DSS in tumor stage III. (D) RFS in tumor stage I. (E) RFS in tumor stage II. (F) RFS in tumor stage III. (G-L) Kaplan-Meier analyses comparing DSS and RFS in patients with luminal B breast cancer between the *SLC20A1*^high^ and *SLC20A1*^low^ groups. (G) DSS in tumor stage I. (H) DSS in tumor stage II. (I) DSS in tumor stage III. (J) RFS in tumor stage I. (K) RFS in tumor stage II. (L) RFS in tumor stage III.

To examine the prognosis of patients with luminal A and luminal B breast cancer at each tumor stage, we next performed Kaplan-Meier analysis to compare DSS and RFS between the *SLC20A1*^high^ and *SLC20A1*^low^ groups at tumor stages I, II or III. Kaplan-Meier analysis showed that patients with *SLC20A1*^high^ luminal A breast cancer at tumor stages I, II and III showed poorer clinical outcomes (DSS: stage I, *p*<0.001; stage II, *p*<0.001; stage III, *p* = 0.030) (RFS: stage I, *p*<0.001; stage II, *p* = 0.0052; stage III, *p* = 0.38) (Fig 2A–2F). On the other hand, there was no significant difference between patients with *SLC20A1*^high^ and *SLC20A1*^low^ luminal B breast cancer at tumor stages I and II (DSS: stage I; *p* = 0.15; stage II, *p* = 0.42) (RFS: stage I, *p* = 0.19; stage II, *p* = 0.071). However, patients with *SLC20A1*^high^ luminal B breast cancer at tumor stage III showed longer survival (DSS: stage III, *p* = 0.027) (RFS: stage III, *p* = 0.21) (Fig 2G–2L). Following Kaplan-Meier analysis, multivariate Cox regression analysis indicated that among the patients with luminal A breast cancer, those at tumor stages I and II in the *SLC20A1*^high^ group exhibited poorer clinical outcomes (DSS: stage I, HR = 3.74, 95% CI = 1.87–7.47; stage II, HR = 2.30, 95% CI = 1.37–3.85; RFS: stage I, HR = 2.88, 95% CI = 1.71–4.86; stage II, HR = 1.99, 95% CI = 1.22–3.26); however, this was not the case, in patients at tumor stage III (DSS: HR = 2.34, 95% CI = 0.53–10.36; RFS: HR = 1.49, 95% CI = 0.52–4.30) (Table 2). By contrast, patients with *SLC20A1*^high^ luminal B breast cancer at tumor stages I and II did not exhibit poorer clinical outcomes (DSS: stage I, HR = 1.67, 95% CI = 0.82–3.40; stage II, HR = 1.22, 95% CI = 0.79–1.87; RFS: stage I, HR = 1.65, 95% CI = 0.76–3.59; stage II, HR = 1.46, 95% CI = 0.96–2.22). However, patients at tumor stage III exhibited longer survival rates (stage III: DSS, HR = 0.31, 95% CI = 0.10–0.93; RFS, HR = 1.63, 95% CI = 0.65–4.11) (Table 2). These results strongly suggest that *SLC20A1* may be applied as a prognostic biomarker for luminal A breast cancer at the early stages.

**Table 2:** Multivariate Cox regression analyses of disease-specific survival (DSS) and relapse-free status (RFS) in patients with luminal A and luminal B breast cancer at tumor stages I, II and III.

	DSS		
	Hazard ratio^a^	95% confidence interval	*P*-value
Luminal A			
**Stage I**	**3.74**	**1.87–7.47**	**< 0.001**
**Stage II**	**2.30**	**1.37–3.85**	**0.0016**
Stage III	2.34	0.53–10.36	0.26
Luminal B			
Stage I	1.67	0.82–3.40	0.16
Stage II	1.22	0.79–1.87	0.38
Stage III	0.31	0.10–0.93	0.036
	RFS		
	Hazard ratio^a^	95% confidence interval	*P*-value
Luminal A			
**Stage I**	**2.88**	**1.71–4.86**	**< 0.001**
**Stage II**	**1.99**	**1.22–3.26**	**0.0060**
Stage III	1.49	0.52–4.30	0.46
Luminal B			
Stage I	1.65	0.76–3.59	0.20
Stage II	1.46	0.96–2.22	0.073
Stage III	1.63	0.65–4.11	0.30

^a^*SLC20A1*^high^ group’s hazard ratio to *SLC20A1*^low^ group adjusted using age as a confounding factor as estimated using Cox proportional hazard model. Significant differences are shown in bold and underlined indicates good prognosis.

### Endocrine therapy is insufficient for *SLC20A1*^high^ luminal A and luminal B breast cancers by Kaplan-Meier analyses and multivariate Cox regression analyses

**Fig 3:**
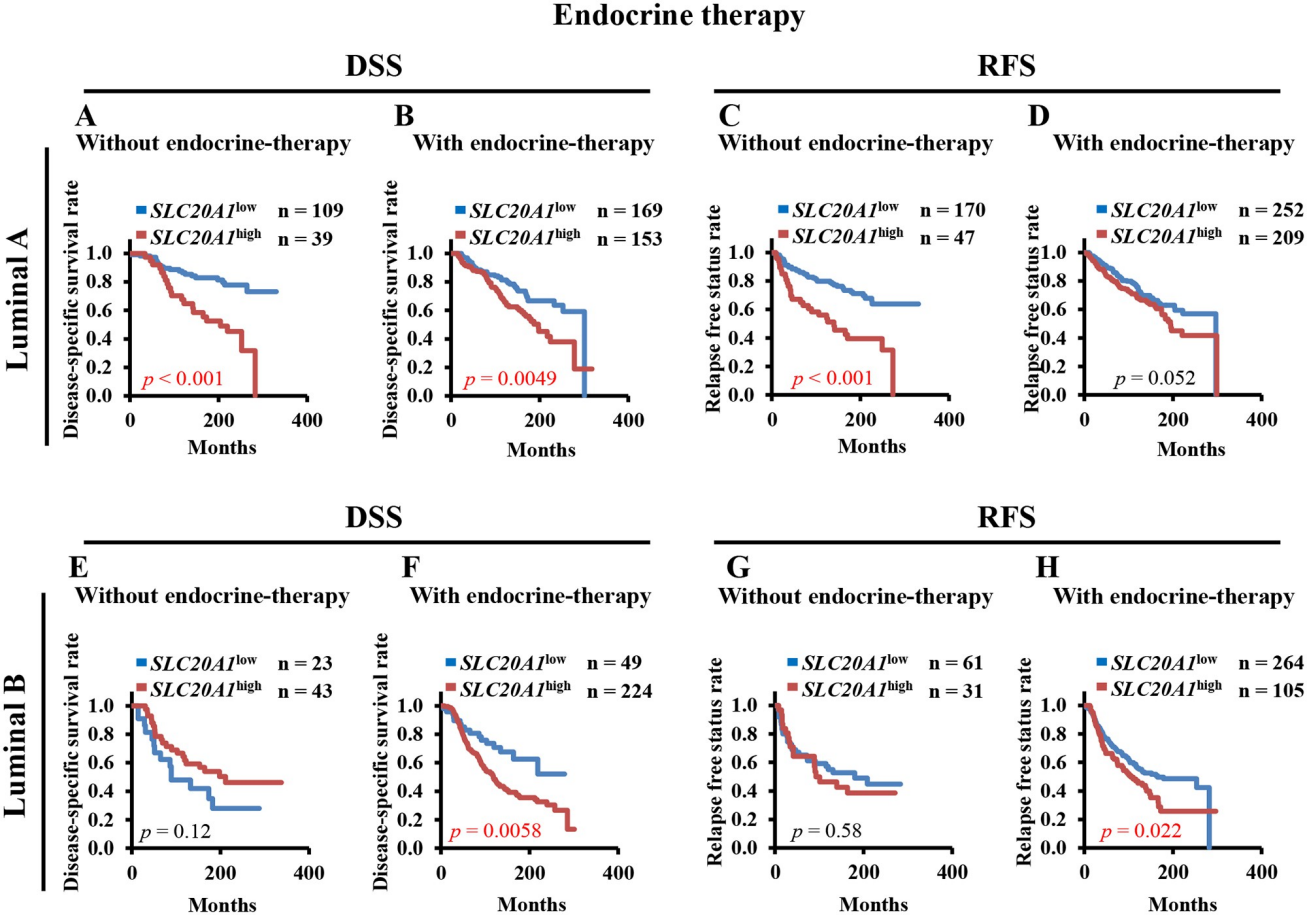
Higher solute carrier family 20 member 1 (SLC20A1) expression (*SLC20A1*^high^) confer poorer clinical outcomes in the luminal A and luminal B breast cancer subtypes following endocrine therapy. (A-D) Kaplan-Meier analyses comparing disease-specific survival (DSS) and relapse-free status (RFS) between the *SLC20A1*^high^ and low SLC20A1 expression (*SLC20A1*^low^) groups in patients with luminal A breast cancer without or with endocrine therapy. (A) DSS in the group without endocrine therapy. (B) DSS in the group with endocrine therapy. (C) RFS in the group without endocrine therapy. (D) RFS in the group with endocrine therapy. (E-H) Kaplan-Meier analyses comparing DSS and RFS between the *SLC20A1*^high^ and *SLC20A1*^low^ groups in patients with luminal B breast cancer without or with endocrine therapy. (E) DSS in the group without endocrine therapy. (F) DSS in the group with endocrine therapy. (G) RFS in the group without endocrine therapy. (H) RFS in the group with endocrine therapy.

We next examined the outcomes of using endocrine therapy as the main treatment for ER+ breast cancer. Chemotherapy is also used to the part of the treatment regimen for luminal A and luminal B breast cancer [2–4, 10, 11]. Patients with luminal A and luminal B breast cancer who received chemotherapy in the analyzed dataset in the present study constituted only luminal A: 8.0% (54/679) and luminal B: 9.9% (47/475) of the population (S1 Table). Therefore, we first performed Kaplan-Meier analysis comparing DSS and RFS between the *SLC20A1*^high^ and *SLC20A1*^low^ groups in patients with luminal A and luminal B breast cancer who did or did not receive endocrine therapy. Patients with *SLC20A1*^high^ luminal A breast cancer showed poorer clinical outcomes (without endocrine therapy: DSS, *p*<0.001; RFS, *p*<0.001) (with endocrine therapy: DSS, *p* = 0.0049; RFS, *p* = 0.052) (Fig 3A–3D). Although patients with *SLC20A1*^high^ luminal B breast cancer who did not receive endocrine therapy did not display poorer clinical outcomes (DSS: *p* = 0.12, RFS, *p* = 0.58) (Fig 3E and 3G), patients who received endocrine therapy showed poorer clinical outcomes (DSS, *p* = 0.0058, RFS, *p* = 0.022) (Fig 3F and 3H). Subsequently, multivariate Cox regression analysis of DSS and RFS was performed with age as a confounding factor. Patients with *SLC20A1*^high^ luminal A breast cancer exhibited poorer clinical outcomes regardless of whether they received endocrine therapy (without endocrine therapy: DSS, HR = 2.94, 95% CI = 1.58–5.48; RFS, HR = 2.86, 95% CI = 1.78–4.59; with endocrine therapy: DSS, HR = 1.60, 95% CI = 1.08–2.38; RFS, HR = 1.36, 95% CI = 0.98–1.88) (Table 3). Patients with *SLC20A1*^high^ luminal B breast cancer without endocrine therapy did not exhibit poorer clinical outcomes (DSS, HR = 0.59, 95% CI = 0.30–1.15; RFS, HR = 1.22, 95% CI = 0.67–2.21). Patients with *SLC20A1*^high^ who received endocrine therapy exhibited poorer clinical outcomes (DSS: HR = 2.09, 95% CI = 1.24–3.52; RFS, HR = 1.44, 95% CI = 1.05–1.97) (Table 3). To conclude, both Kaplan-Meier and multivariate Cox regression analyses suggest that endocrine therapy is insufficient for *SLC20A1*^high^ luminal A and *SLC20A1*^high^ luminal B breast cancer.

**Table 3:** Comparison of disease-specific survival (DSS) and relapse-free status (RFS) in luminal A and luminal B breast cancer without or with endocrine therapy.

	DSS		
	Hazard ratio^a^	95% confidence interval	*P*-value
Without endocrine therapy			
**Luminal A**	**2.94**	**1.58–5.48**	**<0.001**
Luminal B	0.59	0.30–1.15	0.12
With endocrine therapy			
**Luminal A**	**1.60**	**1.08–2.38**	**0.019**
**Luminal B**	**2.09**	**1.24–3.52**	**0.0054**
	RFS		
	Hazard ratio^a^	95% confidence interval	*P*-value
Without endocrine therapy			
**Luminal A**	**2.86**	**1.78–4.59**	**<0.001**
Luminal B	1.22	0.67–2.21	0.52
With endocrine therapy			
Luminal A	1.36	0.98–1.88	0.062
**Luminal B**	**1.44**	**1.05–1.97**	**0.022**

^a^*SLC20A1*^high^ group’s hazard ratio to *SLC20A1*^low^ group adjusted using age as a confounding factor as estimated using Cox proportional hazard model. Significant differences are shown in bold.

### Endocrine therapy at early tumor-stage is insufficient for *SLC20A1*^high^ luminal A type breast cancer by Kaplan-Meier analyses and multivariate Cox regression analyses

**Fig 4:**
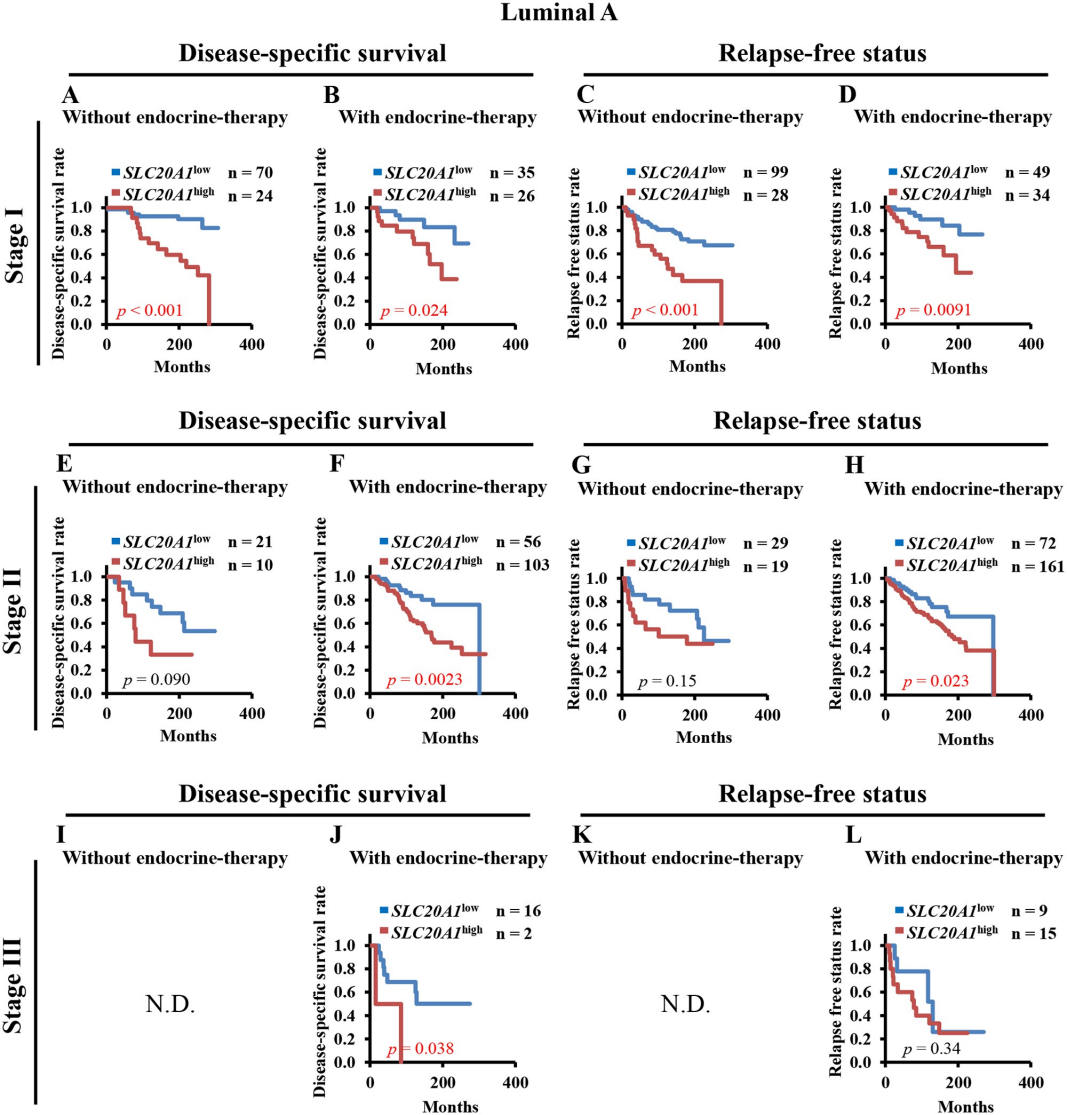
High solute carrier family 20 member 1 (SLC20A1) expression (*SLC20A1*^high^) confer poorer clinical outcomes in patients with luminal A breast cancer at the early stages with endocrine therapy. (A-D) Kaplan-Meier analyses comparing disease-specific survival (DSS) or relapse-free status (RFS) between the *SLC20A1*^high^ and low SLC20A1 expression (*SLC20A1*^low^) groups amongst patients with stage I luminal A breast cancer without or with endocrine therapy. (A) Comparing DSS of patients without endocrine therapy. (B) Comparing DSS of patients with endocrine therapy. (C) Comparing RFS of patients without endocrine therapy. (D) Comparing RFS of patients with endocrine therapy. (E-H) Kaplan-Meier analyses comparing DSS or RFS between the *SLC20A1*^high^ and *SLC20A1*^low^ groups amongst patients with stage II luminal A breast cancer without or with endocrine therapy. (E) Comparing DSS of patients without endocrine therapy, (F) Comparing DSS of patients with endocrine therapy, (G) Comparing RFS of patients without endocrine therapy, (H) Comparing RFS of patients with endocrine therapy. (I-L) Kaplan-Meier analyses comparing DSS or RFS between the *SLC20A1*^high^ and *SLC20A1*^low^ groups amongst patients with stage III luminal A breast cancer without or with endocrine therapy. (I) Comparing DSS of patients without endocrine therapy. (J) Comparing DSS of patients with endocrine therapy. (K) Comparing RFS of patients without endocrine therapy. (L) Comparing RFS of patients with endocrine therapy. (I and K) There were insufficient numbers for Kaplan-Meier analysis. N.D. stands for “not determined”.

**Fig 5:**
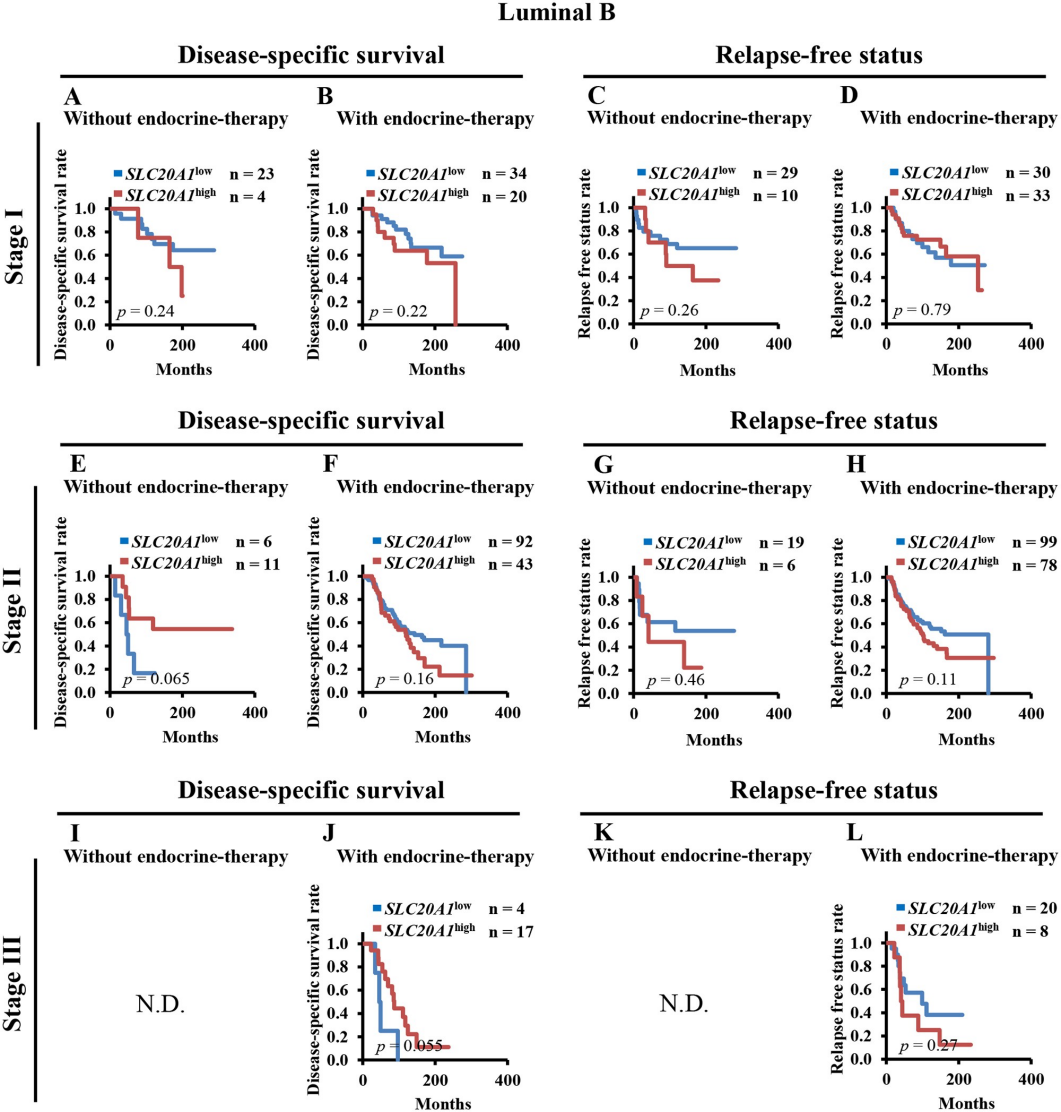
There are no significant differences between high solute carrier family 20 member 1 (SLC20A1) expression (*SLC20A1*^high^) and low SLC20A1 expression (*SLC20A1*^low^) in patients with luminal B breast cancer at each tumor stage undergoing endocrine therapy. (A-D) Kaplan-Meier analyses comparing disease-specific survival (DSS) or relapse-free status (RFS) between the *SLC20A1*^high^ and *SLC20A1*^low^ groups amongst patients with stage I luminal B breast cancer without or with endocrine therapy. (A) Comparing DSS of patients without endocrine therapy. (B) Comparing DSS of patients with endocrine therapy. (C) Comparing RFS of patients without endocrine therapy. (D) Comparing RFS of patients with endocrine therapy. (E-H) Kaplan-Meier analyses comparing DSS or RFS between the *SLC20A1*^high^ and *SLC20A1*^low^ groups amongst patients with stage II luminal B breast cancer without or with endocrine therapy. (E) Comparing DSS of patients without endocrine therapy. (F) Comparing DSS of patients with endocrine therapy, (G) Comparing RFS of patients without endocrine therapy. (H) Comparing RFS of patients with endocrine therapy. (I-L) Kaplan-Meier analyses comparing DSS or RFS between the *SLC20A1*^high^ and *SLC20A1*^low^ groups amongst patients with stage III luminal B breast cancer without or with endocrine therapy. (I) Comparing DSS of patients without endocrine therapy, (J) Comparing DSS of patients with endocrine therapy, (K) Comparing RFS at patients without endocrine therapy, (L) Comparing RFS of patients with endocrine therapy. (I and K) There were insufficient numbers for Kaplan-Meier analysis. N.D. stands for “not determined”.

To determine the relationship between the prognoses of patients with *SLC20A1*^high^ and endocrine therapy at each tumor stage, we performed Kaplan-Meier analysis of DSS and RFS between the *SLC20A1*^high^ and *SLC20A1*^low^ groups for both patients with luminal A and luminal B breast cancer without or with endocrine therapy at tumor stages I, II or III. At tumor stage I, patients with *SLC20A1*^high^ luminal A breast cancer showed poor clinical outcomes regardless of whether they received endocrine therapy (without endocrine therapy: DSS, *p*<0.001, RFS, *p*<0.001) (with endocrine therapy: DSS, *p* = 0.024, RFS, *p* = 0.0091) (Fig 4A–4D). At tumor stage II, although patients with luminal A breast cancer who did not receive endocrine therapy in the *SLC20A1*^high^ group did not show poorer clinical outcomes (DSS, *p* = 0.090, RFS, *p* = 0.15) (Fig 4E and 4G), those with the *SLC20A1*^high^ luminal A type with endocrine therapy showed poorer clinical outcomes (DSS, *p* = 0.0023, RFS, *p* = 0.023) (Fig 4F and 4H). At tumor stage III, there were insufficient numbers of patients with luminal A breast cancer who did not receive endocrine therapy for analysis (Fig 4I and 4K). At tumor stage III, patients with *SLC20A1*^high^ luminal A breast cancer who underwent endocrine therapy showed shorter survival (DSS, *p* = 0.038, RFS, *p* = 0.34) (Fig 4J and 4L). By contrast, amongst patients with the luminal B subtype at tumor stage I, none of those in the *SLC20A1*^high^ group exhibited poor clinical outcomes (without endocrine therapy: DSS, *p* = 0.24, RFS, *p* = 0.26; with endocrine therapy: DSS, *p* = 0.22, RFS, *p* = 0.79) (Fig 5A–5D). At tumor stages II, none of the patients with *SLC20A1*^high^ luminal B breast cancer also exhibited poor clinical outcomes, regardless of whether they received endocrine therapy (without endocrine therapy: DSS, *p* = 0.065, RFS, *p* = 0.46; with endocrine therapy: DSS, *p* = 0.16, RFS, *p* = 0.11) (Fig 5E–5H). At tumor stage III, there were insufficient numbers of patients with not only luminal A, but also luminal B breast cancer who did not receive endocrine therapy for analysis (Fig 5I and 5K). Patients with *SLC20A1*^high^ luminal B breast cancer did not exhibit poorer clinical outcomes (DSS, *p* = 0.055, RFS, *p* = 0.27) (Fig 5J and 5L).

Subsequently, multivariate Cox regression analyses of DSS and RFS was performed. The results revealed similar results to the aforementioned Kaplan-Meier analyses. At tumor stage I, patients with *SLC20A1*^high^ luminal A breast cancer exhibited poor clinical outcomes regardless of endocrine therapy (without endocrine therapy: DSS, HR = 5.57, 95% CI = 2.20–14.11; RFS, HR = 3.10, 95% CI = 1.65–5.81; with endocrine therapy: DSS, HR = 3.55, 95% CI = 1.17–10.81; RFS, HR = 3.46, 95% CI = 1.28–9.34) (Table 4). Patients with *SLC20A1*^high^ luminal B breast cancer did not exhibit poor clinical outcomes in an endocrine therapy-independent manner (without endocrine therapy: DSS, HR = 3.13, 95% CI = 0.69–14.25; RFS, HR = 2.50, 95% CI = 0.81–7.68; with endocrine therapy: DSS, HR = 1.89, 95% CI = 0.73–4.88; RFS, HR = 0.82, 95% CI = 0.37–1.85) (Table 4). At tumor stage II, although patients with *SLC20A1*^high^ luminal A breast cancer did not exhibit poor clinical outcomes even if they did not undergo endocrine therapy, patients with *SLC20A1*^high^ luminal A breast cancer who underwent endocrine therapy exhibited poorer clinical outcomes (without endocrine therapy: DSS, HR = 2.42, 95% CI = 0.82–7.18; RFS, HR = 1.61, 95% CI = 0.63–4.14) (with endocrine therapy: DSS, HR = 2.64, 95% CI = 1.36–5.12; RFS, HR = 1.83, 95% CI = 1.07–3.14) (Table 4). Similar to patients at tumor stage I, patients with *SLC20A1*^high^ luminal B breast cancer at tumor stage II did not exhibit poor clinical outcomes in an endocrine therapy-independent manner (without endocrine therapy: DSS, HR = 0.26, 95% CI = 0.07–0.94; RFS, HR = 1.44, 95% CI = 0.40–5.14; with endocrine therapy: DSS, HR = 1.32, 95% CI = 0.82–2.12; RFS, HR = 1.40, 95% CI = 0.91–2.15) (Table 4). At tumor stage III, there were insufficient numbers of patients with luminal A or luminal B breast cancer who did not receive endocrine therapy for analysis. At this stage, patients with *SLC20A1*^high^ luminal A and B breast cancer who underwent endocrine therapy did not exhibit poor clinical outcomes (luminal A: DSS, HR = 1.98, 95% CI = 0.35–11.32; RFS, HR = 1.68, 95% CI = 0.52–5.38; luminal B: DSS, HR = 0.35, 95% CI = 0.10–1.15; RFS, HR = 1.71, 95% CI = 0.63–4.64) (Table 4). Taken together, these results suggest that the administration of endocrine therapy beginning from the early tumor stages is less effective for patients with *SLC20A1*^high^ luminal A breast cancer.

**Table 4:** Multivariate Cox regression analyses of disease-specific survival (DSS) and relapse-free status (RFS) in patients with luminal A and luminal B without or with endocrine therapy at various tumor stages.

	DSS		
	Hazard ratio^a^	95% confidence interval	*P*-value
Stage I			
Without endocrine therapy			
**Luminal A**	**5.57**	**2.20–14.11**	**<0.001**
Luminal B	3.13	0.69–14.25	0.14
With endocrine therapy			
**Luminal A**	**3.55**	**1.17–10.81**	**0.026**
Luminal B	1.89	0.73–4.88	0.19
Stage II			
Without endocrine therapy			
Luminal A	2.42	0.82–7.18	0.11
Luminal B	0.26	0.07–0.94	0.041
With endocrine therapy			
**Luminal A**	**2.64**	**1.36–5.12**	**0.0040**
Luminal B	1.32	0.82–2.12	0.26
Stage III			
Without endocrine therapy			
Luminal A	N.D.	N.D.	N.D.
Luminal B	N.D.	N.D.	N.D.
With endocrine therapy			
Luminal A	1.98	0.35–11.32	0.44
Luminal B	0.35	0.10–1.15	0.083
	RFS		
	Hazard ratio^a^	95% confidence interval	*P*-value
Stage I			
Without endocrine therapy			
**Luminal A**	**3.10**	**1.65–5.81**	**<0.001**
Luminal B	2.50	0.81–7.68	0.11
With endocrine therapy			
**Luminal A**	**3.46**	**1.28–9.34**	**0.014**
Luminal B	0.82	0.37–1.85	0.64
Stage II			
Without endocrine therapy			
Luminal A	1.61	0.63–4.14	0.32
Luminal B	1.44	0.40–5.14	0.57
With endocrine therapy			
**Luminal A**	**1.83**	**1.07–3.14**	**0.027**
Luminal B	1.40	0.91–2.15	0.12
stage III			
Without endocrine therapy			
Luminal A	N.D.	N.D.	N.D.
Luminal B	N.D.	N.D.	N.D.
With endocrine therapy			
Luminal A	1.68	0.52–5.38	0.38
Luminal B	1.71	0.63–4.64	0.30

^a^*SLC20A1*^high^ group’s hazard ratio to *SLC20A1*^low^ group adjusted using age as a confounding factor as estimated using Cox proportional hazard model. Significant differences are shown in bold and underlined indicates good prognosis.

### Chemotherapy is an effective treatment option for patients with *SLC20A1*^high^ luminal A and B breast cancer according to Kaplan-Meier analysis and multivariate Cox regression analysis

**Fig 6:**
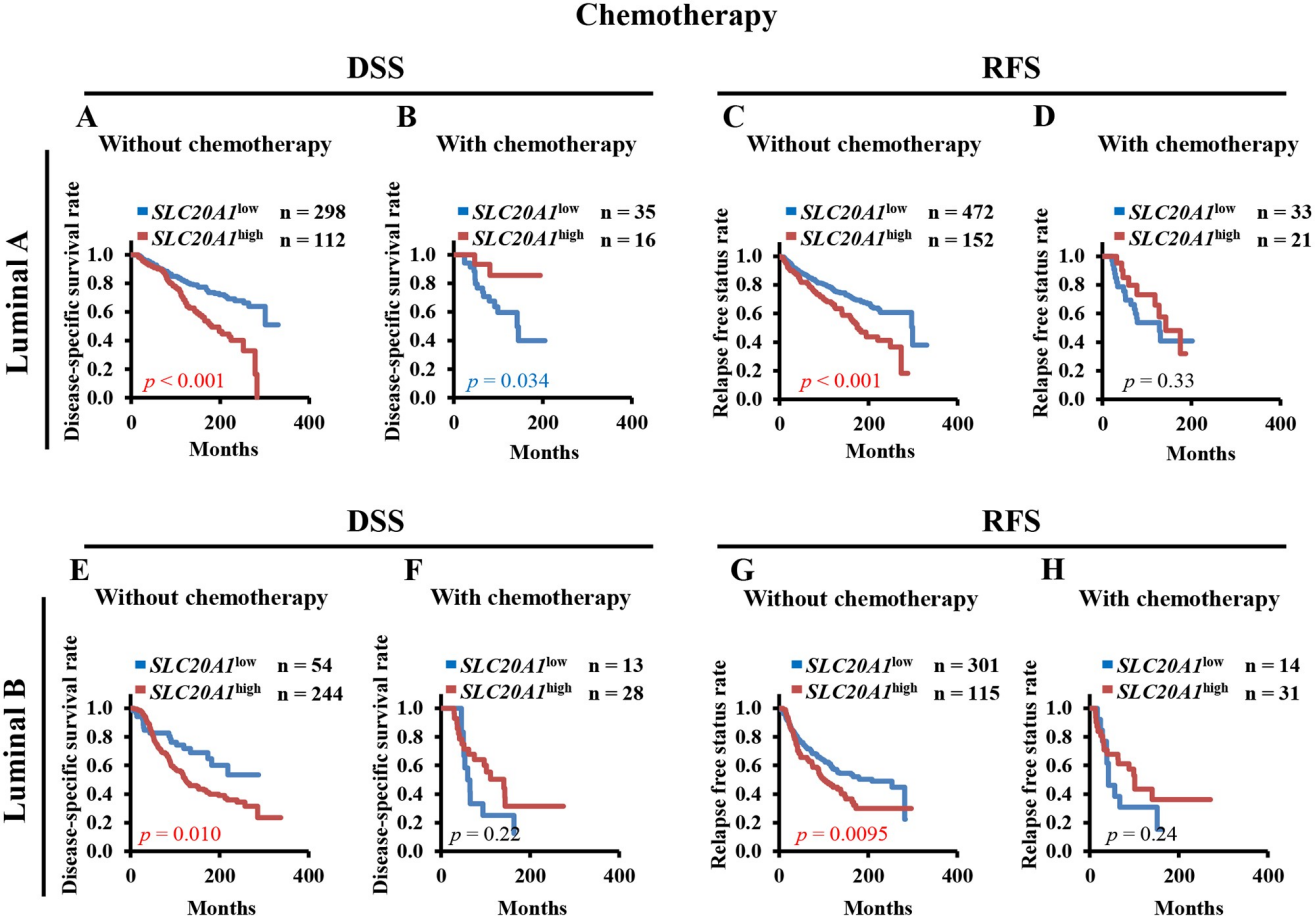
High solute carrier family 20 member 1 (SLC20A1) expression (*SLC20A1*^high^) does not confer poor clinical outcomes in the luminal A and luminal B subtypes following chemotherapy. (A-D) Kaplan-Meier analyses comparing disease-specific survival (DSS) and relapse-free status (RFS) between the *SLC20A1*^high^ and low SLC20A1 expression (*SLC20A1*^low^) groups amongst patients with luminal A breast cancer without or with chemotherapy. (A) DSS in the group without chemotherapy. (B) DSS in the group with chemotherapy. (C) RFS in the group without chemotherapy. (D) RFS in the group with chemotherapy. (E-H) Kaplan-Meier analyses comparing the DSS or RFS between the *SLC20A1*^high^ and *SLC20A1*^low^ groups amongst patients with luminal B breast cancer without or with chemotherapy. (E) DSS in the group without chemotherapy. (F) DSS in the group with chemotherapy. (G) RFS in the group without chemotherapy. (H) RFS in the group with chemotherapy.

Although there was a relatively small number of chemotherapy cases in the present study, chemotherapy was selected as the treatment option for ER+ breast cancer (luminal A: DSS, n = 51; RFS; n = 54) (luminal B: DSS, n = 41; RFS, n = 44). In addition, we previously reported that chemotherapy was sufficient for *SLC20A1*^high^ claudin-low and basal-like breast cancers [25]. Therefore, for the present study we also classified patients with luminal A or luminal B into the without and with chemotherapy categories before performing Kaplan-Meier and multivariate Cox regression analyses of DSS and RFS. Kaplan-Meier analysis revealed that patients with *SLC20A1*^high^ luminal A and luminal B breast cancer who did not undergo chemotherapy showed poorer clinical outcomes (luminal A: DSS, *p*<0.001, RFS, *p*<0.001) (luminal B: DSS, *p* = 0.010, RFS, *p* = 0.0095) (Fig 6A, 6C, 6E and 6G). Interestingly, patients with *SLC20A1*^high^ luminal A and luminal B breast cancer who underwent chemotherapy did not show poor clinical outcomes (luminal A: DSS, *p =* 0.034, RFS, *p* = 0.33) (luminal B: DSS, *p* = 0.22, RFS, *p* = 0.24) (Fig 6B, 6D, 6F and 6H).

Multivariate Cox regression analysis demonstrated that patients with *SLC20A1*^high^ luminal A and luminal B breast cancer without chemotherapy showed poorer clinical outcomes (luminal A: DSS, HR = 2.00, 95% CI = 1.41–2.85; RFS, HR = 1.79, 95% CI = 1.33–2.40) (luminal B: DSS, HR = 1.96, 95% CI = 1.20–3.20; RFS, HR = 1.48, 95% CI = 1.10–1.99) (Table 5). According to Kaplan-Meier analysis, patients with *SLC20A1*^high^ luminal A and luminal B breast cancer with chemotherapy did not show poor clinical outcomes (luminal A: DSS, HR = 0.30, 95% CI = 0.066–1.35; RFS, HR = 0.72, 95% CI = 0.31–1.65) (luminal B: DSS, HR = 0.61, 95% CI = 0.27–1.35; RFS, HR = 0.65, 95% CI = 0.29–1.43) (Table 5). Taken together, these results suggest that chemotherapy is an effective treatment option for patients with *SLC20A1*^high^ luminal A or luminal B.

**Table 5:** Multivariate Cox regression analyses of disease-specific survival (DSS) or relapse-free status (RFS) in patients with luminal A and luminal B breast cancer without/with chemotherapy.

	DSS		
	Hazard ratio^a^	95% confidence interval	*P*-value
Without chemotherapy			
**Luminal A**	**2.00**	**1.41–2.85**	**<0.001**
**Luminal B**	**1.96**	**1.20–3.20**	**0.0074**
With chemotherapy			
Luminal A	0.30	0.066–1.35	0.12
Luminal B	0.61	0.27–1.35	0.22
	RFS		
	Hazard ratio^a^	95% confidence interval	*P*-value
Without chemotherapy			
**Luminal A**	**1.79**	**1.33–2.40**	**<0.001**
**Luminal B**	**1.48**	**1.10–1.99**	**0.0098**
With chemotherapy			
Luminal A	0.72	0.31–1.65	0.44
Luminal B	0.65	0.29–1.43	0.28

^a^*SLC20A1*^high^ group’s hazard ratio to *SLC20A1*^low^ group adjusted using age as a confounding factor as estimated using Cox proportional hazard model. Significant differences are shown in bold.

### Patients with *SLC20A1*^high^ luminal A or luminal B breast cancer who underwent endocrine therapy are at higher risk of late recurrence

Patients with luminal A or luminal B breast cancer typically exhibit superior prognoses compared with other subtypes breast cancer [7–12]. However, some patients with the luminal A and luminal B subtypes will relapse after the termination of long-term endocrine therapy [14–21]. Therefore, late recurrence is one of the key clinical issues of luminal A and luminal B breast cancer that needs to be addressed following endocrine therapy. Kaplan-Meier analyses of RFS showed marked differences between the *SLC20A1*^high^ and *SLC20A1*^low^ groups from 175 months onwards (Fig 3D and 3H).

**Fig 7:**
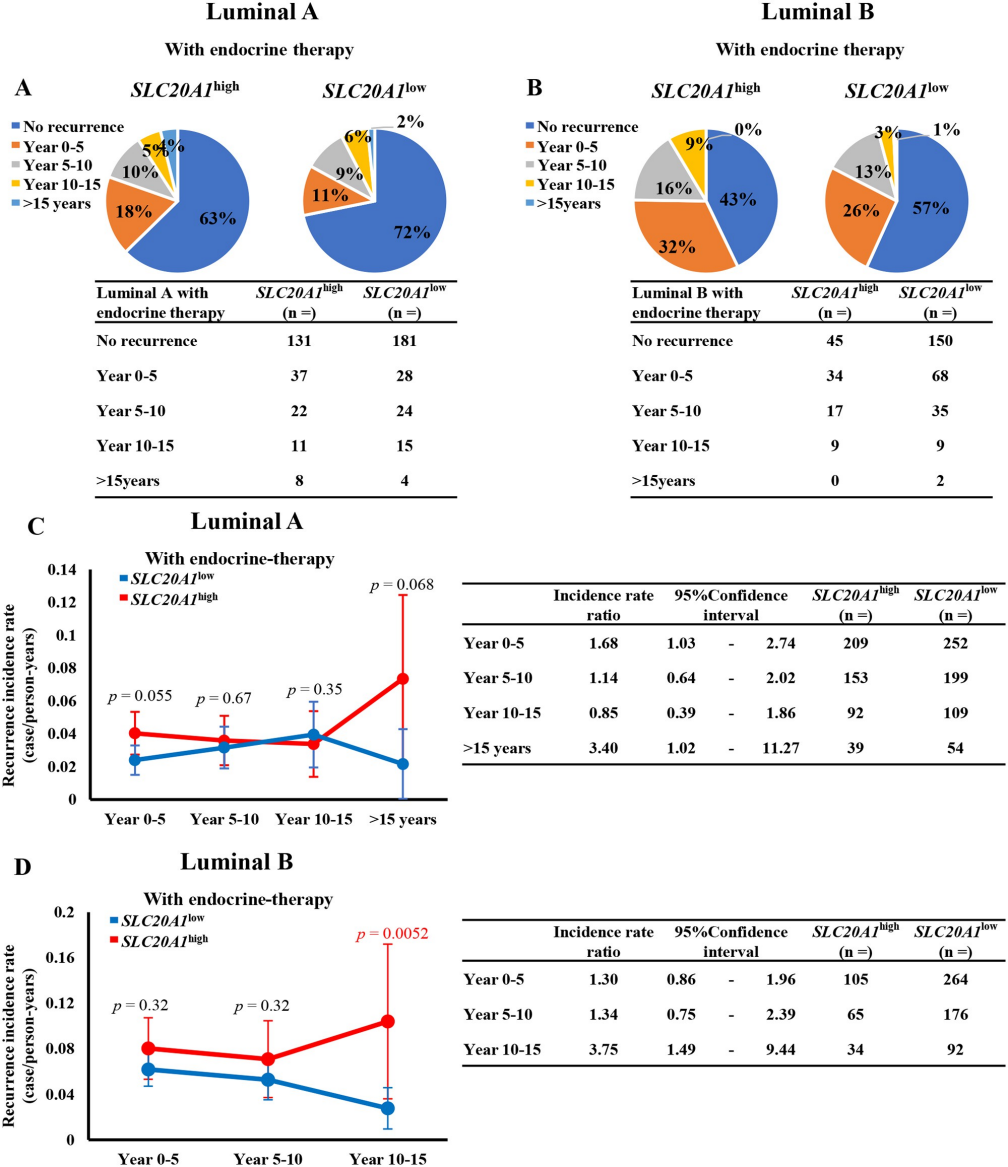
High solute carrier family 20 member 1 (SLC20A1) expression (*SLC20A1*^high^) confers higher recurrence incidence rates compared with patients with low SLC20A1 expression (*SLC20A1*^low^) after long periods of time in both luminal A and luminal B subtypes. (A-B) Pie charts of the recurrent period and number of patients in the *SLC20A1*^high^ and *SLC20A1*^low^ groups amongst those with luminal A or luminal B breast cancer. (A) Patients with *SLC20A1*^high^ (left) and *SLC20A1*^low^ (right) luminal A. (B) Patients with *SLC20A1*^high^ (left) and *SLC20A1*^low^ (right) luminal B. (C-D) Line graphs showing the recurrence incidence rates of patients in the *SLC20A1*^high^ and *SLC20A1*^low^ luminal A or luminal B groups every 5 years with or without endocrine therapy. *P*-values were calculated using the statistic based on normal distribution and corrected using the Holm method. The incidence rate ratio was calculated as the ratio of the recurrence incidence rate of *SLC20A1*^high^ to that of *SLC20A1*^low^. The 95% confidence interval and the number of patients were shown on the right sides. (C) Patients with luminal A. (D) Patients with luminal B.

Calculations of the recurrent period and number of patients in the *SLC20A1*^high^ and *SLC20A1*^low^ groups amongst those with luminal A or luminal B breast cancer are shown (Fig 7A and 7B). We next analyzed the recurrence incidence rate and the rate ratio every 5 years from the time of diagnosis. Amongst patients with luminal A breast cancer who underwent endocrine therapy, those in the *SLC20A1*^high^ group showed similar recurrence incidence rates compared with those in the *SLC20A1*^low^ group at year 0–5, year 5–10 and year 10–15 (year 0–5: Incidence rate ratio = 1.68, 95% CI = 1.03–2.74) (year 5–10: Incidence rate ratio = 1.14, 95% CI = 0.64–2.02) (year 10–15: Incidence rate ratio = 0.85, 95% CI = 0.39–1.86). In particular, patients with *SLC20A1*^high^ showed higher recurrence incidence rates compared with those with *SLC20A1*^low^ at >15 years (Incidence rate ratio = 3.40, 95% CI = 1.02–11.27) (Fig 7C).

Amongst patients with luminal B breast cancer who received endocrine therapy, those in the *SLC20A1*^high^ group showed similar recurrence incidence rates compared with those in the *SLC20A1*^low^ group at year 0–5 and year 5–10 (year 0–5: Incidence rate ratio = 1.30, 95% CI = 0.86–1.96) (year 5–10; Incidence rate ratio = 1.34, 95% CI = 0.75–2.39). Furthermore, patients in the *SLC20A1*^high^ group showed higher recurrence incidence rates compared with those in the *SLC20A1*^low^ group at year 10–15 (Incidence rate ratio = 3.75, 95% CI = 1.49–9.44). Since none of patients with *SLC20A1*^high^ recurred at >15 years, the incidence rate ratio could not be analyzed for this time period (Fig 7D). To conclude, these results suggest that patients with *SLC20A1*^high^ exhibit higher risk of late recurrence even with endocrine therapy.

## Discussion

Patients with early-stage *SLC20A1*^high^ luminal A breast cancer showed poorer clinical outcomes in terms of both DSS and RFS (Fig 2A–2F and Table 2), suggesting that *SLC20A1*^high^ can be applied as a viable prognostic biomarker for early-stage luminal A breast cancer. By contrast, patients with *SLC20A1*^high^ luminal B tumors did not show poorer clinical outcomes (Fig 2G–2L and Table 2). Our previous study reported that *SLC20A1*^high^ HER2-enriched subtypes have superior clinical outcomes [25]. Since the luminal B type expresses HER2 strongly, differential HER2 status may be the reason for the different prognoses between the luminal A and luminal B subtypes with *SLC20A1*^high^.

As shown in Fig 3 and Table 3, endocrine therapy for the luminal A and luminal B subtypes of patients with *SLC20A1*^high^ is insufficient for the improvement of prognosis or lengthening of the interval prior to relapse. In the luminal A subtype, patients in the *SLC20A1*^high^ group with endocrine therapy showed poorer prognosis according to DSS, but they did not show significant differences in terms of the recurrence compared with that in the *SLC20A1*^low^ group until 175 months onwards (14.6 years) (Fig 3D). These results suggest that patients with *SLC20A1*^*high*^ are at higher risk of mortality among patients who relapsed ≤175 months regardless of whether *SLC20A1* expression is high or low. Subsequently, we also analyzed the recurrence incidence rate every 5 years. One of the key clinical issues for treating ER+ breast cancer is late recurrence after the end of long-termed endocrine therapy. Therefore, it would be beneficial if the prediction of late recurrence after a long period of therapy can be achieved at the time of diagnosis before the medical treatment commences. Although not statistically significant, patients with the luminal A subtype, *SLC20A1*^high^ were found to be at high risk of recurrence over 15 years (Fig 7C). Among patients with the luminal B subtype, *SLC20A1*^high^ combined with endocrine therapy showed poorer prognoses and shorter intervals to recurrence. This suggests that patients with *SLC20A1*^high^ have higher risks of late recurrence if they received endocrine therapy for year 10–15 (Fig 7D). The difference in RFS observed between the luminal A and luminal B subtypes may be due to differences in the late recurrence rate. In addition, a significant proportion breast cancer cells in the luminal B subtype is HER2-positive. The reason for the difference in relapse rates between the luminal A and luminal B subtypes warrants further study.

In stage I luminal A tumors, patients with *SLC20A1*^high^ who underwent endocrine therapy showed poorer prognoses and shorter intervals to recurrence. By contrast, patients with luminal A who received chemotherapy showed good clinical outcomes. These results indicate that endocrine therapy for patients with stage I *SLC20A1*^high^ luminal A is less effective, such that other treatment options, such as chemotherapy, would be necessary.

It has been proposed that the main cause of late recurrence is dormancy [22, 23]. Dormancy of a cell is defined by the extension of the G_0_/G_1_ phase and cell cycle arrest [22, 23, 35, 36]. SLC20A1 is a Pi symporter, whereas Pi is necessary for DNA synthesis and ATP generation. SLC20A1 depletion has been found to impair cell cycle progression and cell proliferation, in addition to causing cell death [28, 37, 38]. In addition, SLC20A1 siRNA knockdown has been reported to reduce cell viability in MCF7 cells, which was derived from the luminal A subtype of breast cancer [25]. Therefore, it is entirely possible that SLC20A1 overexpression can increase Pi supply into the cell cycle-arrested cells, leading to survival from endocrine therapy and therefore late recurrence. Dormancy has been reported to be one main feature of cancer stem cells (CSCs) [22, 35, 36]. We have previously shown that SLC20A1 knockdown suppressed tumor sphere formation by aldehyde dehydrogenase 1-positive CSCs from claudin-low and basal-like type breast cancer [25]. Therefore, SLC20A1 may contribute to the maintenance of dormant CSCs and lead to late recurrence in luminal A and luminal B breast cancer. By contrast, the suppression of cell proliferation in HeLa cells by SLC20A1 inhibition was found to be independent of Pi uptake [28]. Therefore, the detailed molecular mechanism underlying the effects of SLC20A1^high^ on late recurrence in ER+ breast cancer require further study. SLC20A1^high^ may reduce the efficacy of endocrine therapy against ER+ breast cancer from early tumor stages onwards with regards to late recurrence.

The present study analyzed the METABRIC dataset. To validate the results, another seven breast cancer datasets containing mRNA data were downloaded from cBioportal. All these datasets did not include data of endocrine therapy. Amongst these datasets, TCGA PanCancer Atlas included information of 1,084 patients with DSS, DFS, PFS and staging; however, the average observation period (DSS, 40.8 months; DFS, 37.9 months; PFS, 37.9 months) (S3 Table) was shorter than that in the METABRIC dataset (DSS, 123.6 months; RFS, 110.2 months) (S1 Table). Therefore, TCGA PanCancer Atlas dataset was only used to examine DSS, DFS and PFS, tumor-stage and breast cancer subtypes using Kaplan-Meier and the multivariate Cox regression analyses between the patients in the *SLC20A1*^high^ and *SLC20A1*^low^ groups (S2 and S3 Figs, and S4 Table). The patients with stage II luminal A breast cancer in the *SLC20A1*^high^ group exhibited a tendency towards poor clinical outcomes; this was observed in TCGA PanCancer Atlas dataset, as well as in the METABRIC dataset (S2 Fig). In luminal B breast cancer, TCGA PanCancer Atlas dataset did not reveal similar results to the results of the METABRIC dataset (S3 Fig). This discrepancy between the analyzed results of both cohorts may be due to the smaller number of patients, more censoring, and a shorter observation period in TCGA PanCancer Atlas dataset compared to the METABRIC dataset. The validation of the results of the present study needs to be performed in future. Apart from breast cancer, it has been reported that a high SLC20A1 expression is associated with poor prognoses in pancreatic cancer using Kaplan-Meier and COX hazards analyses of overall survival [39, 40]. Therefore, it is also crucial to perform similar analyses of SLC20A1 in the cohorts of other types of cancer.

Since SLC20A1^high^ was identified to be a prognostic marker using information-theoretical analysis [24], this informative approach may become a powerful tool for identifying novel biomarkers to predict clinical effects at the early stages, especially of late recurrence, in a variety of cancers from the genomic database.

## Conclusions

In the present study, we revealed that patients with *SLC20A1*^high^ showed poorer clinical outcomes at early tumor stages and tend to be less responsive to endocrine therapy for luminal A and luminal B breast cancer. However, patients with *SLC20A1*^high^ who underwent chemotherapy showed good clinical outcomes. In addition, patients with *SLC20A1*^high^ at the time of diagnosis showed higher recurrence incidence rates compared with those in the *SLC20A1*^low^ group at >15 years in those with luminal A and at 10–15 years in those with luminal B. Therefore, *SLC20A1*^high^ can be used as a prognostic biomarker for predicting the effect of endocrine therapy and the likelihood of late recurrence in ER+ breast cancer.

## Supporting information

S1 Fig Expression level of solute carrier family 20 member 1 (SLC20A1) at each stage. (A-C) Box graphs of showing the expression levels of *SLC20A1* at each tumor stage. (A) All stages. (B) Luminal A. (C) Luminal B.
(TIF)

S2 Fig Expression level of solute carrier family 20 member 1 (SLC20A1) at each stage. Kaplan-Meier analyses of patients with luminal A breast cancer with a high solute carrier family 20 member 1 (SLC20A1) expression (SLC20A1^high^) and a low SLC20A1 expression (SLC20A1^low^) at each stage. (A-F) Kaplan-Meier analyses comparing disease-specific survival (DSS), disease-free status (DFS) and progression-free status (PFS) in patients with luminal A breast cancer between the *SLC20A1*^high^ and *SLC20A1*^low^ groups. (A) DSS in tumor stage I. (B) DSS in tumor stage II. (C) DSS in tumor stage III. (D) DFS in tumor stage I. (E) DFS in tumor stage II. (F) DFS in tumor stage III. (G) PFS in tumor stage I. (H) PFS in tumor stage II. (I) PFS in tumor stage III.
(TIF)

S3 Fig Expression level of solute carrier family 20 member 1 (SLC20A1) at each stage. Kaplan-Meier analyses of patients with luminal B breast cancer with a high solute carrier family 20 member 1 (SLC20A1) expression (*SLC20A1*^high^) and a low SLC20A1 expression (*SLC20A1*^low^) at each stage. (A-F) Kaplan-Meier analyses comparing disease-specific survival (DSS), disease-free status (DFS) and progression-free status (PFS) in patients with luminal B breast cancer between the *SLC20A1*^high^ and *SLC20A1*^low^ groups. (A) DSS in tumor stage I. (B) DSS in tumor stage II. (C) DSS in tumor stage III. (D) DFS in tumor stage I. (E) DFS in tumor stage II. (F) DFS in tumor stage III. (G) PFS in tumor stage I. (H) PFS in tumor stage II. (I) PFS in tumor stage III.
(TIF)

S1 Table The number of all, luminal A and luminal B breast cancer clinicopathological data from the METABRIC dataset. (TIF)

S2 Table Youden’s index calculated by receiver operating characteristic (ROC) analysis in each group from the METABRIC dataset. (TIF)

S3 Table The number of all, luminal A and luminal B breast cancer clinicopathological data from TCGA PanCancer Atlas dataset. (TIF)

S4 Table Multivariate Cox regression analyses of disease-specific survival (DSS), disease-free status (DFS) or progression-free status (PFS) in patients with luminal A and luminal B breast cancer at each stage using age as a confounding factor from TCGA PanCancer Atlas dataset. (TIF)

S5 Table Youden’s index calculated using receiver operating characteristic (ROC) analysis in each group from TCGA PanCancer Atlas dataset. (TIF)

S1 Text TCGA PanCancer Atlas dataset analyses. (DOCX)
