# Supplementary figures and images for: High expression of SLC20A1 is less effective for endocrine therapy and predicts late recurrence in ER-positive breast cancer
Source: PLoS One. 2022 May 23;17(5):e0268799. doi: 10.1371/journal.pone.0268799 (PMC9126382; doi:10.1371/journal.pone.0268799)

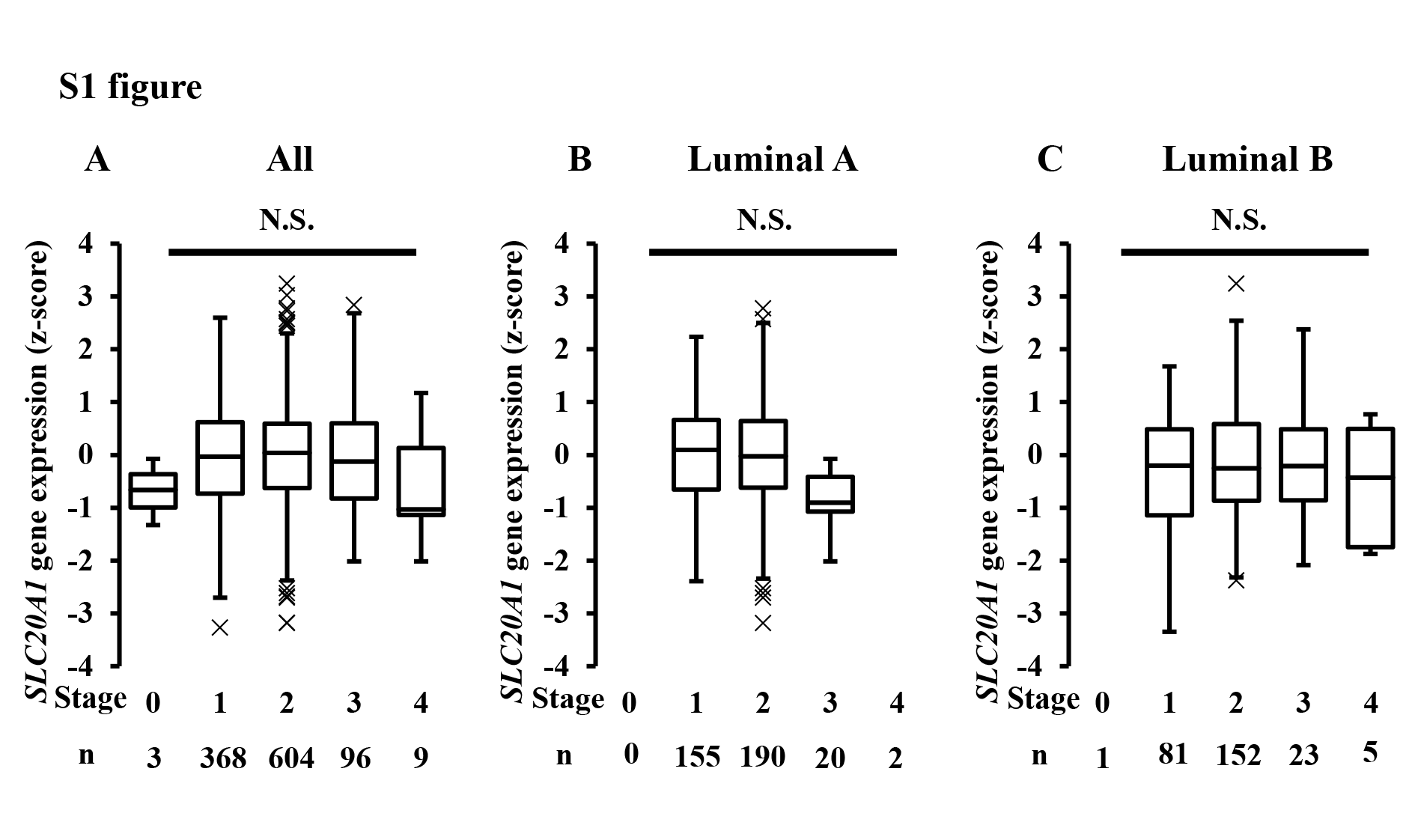

Supplement: S1 Fig — (A-C) Box graphs of showing the expression levels of SLC20A1 at each tumor stage. (A) All stages. (B) Luminal A. (C) Luminal B. (TIF) [file pone.0268799.s001.tif]

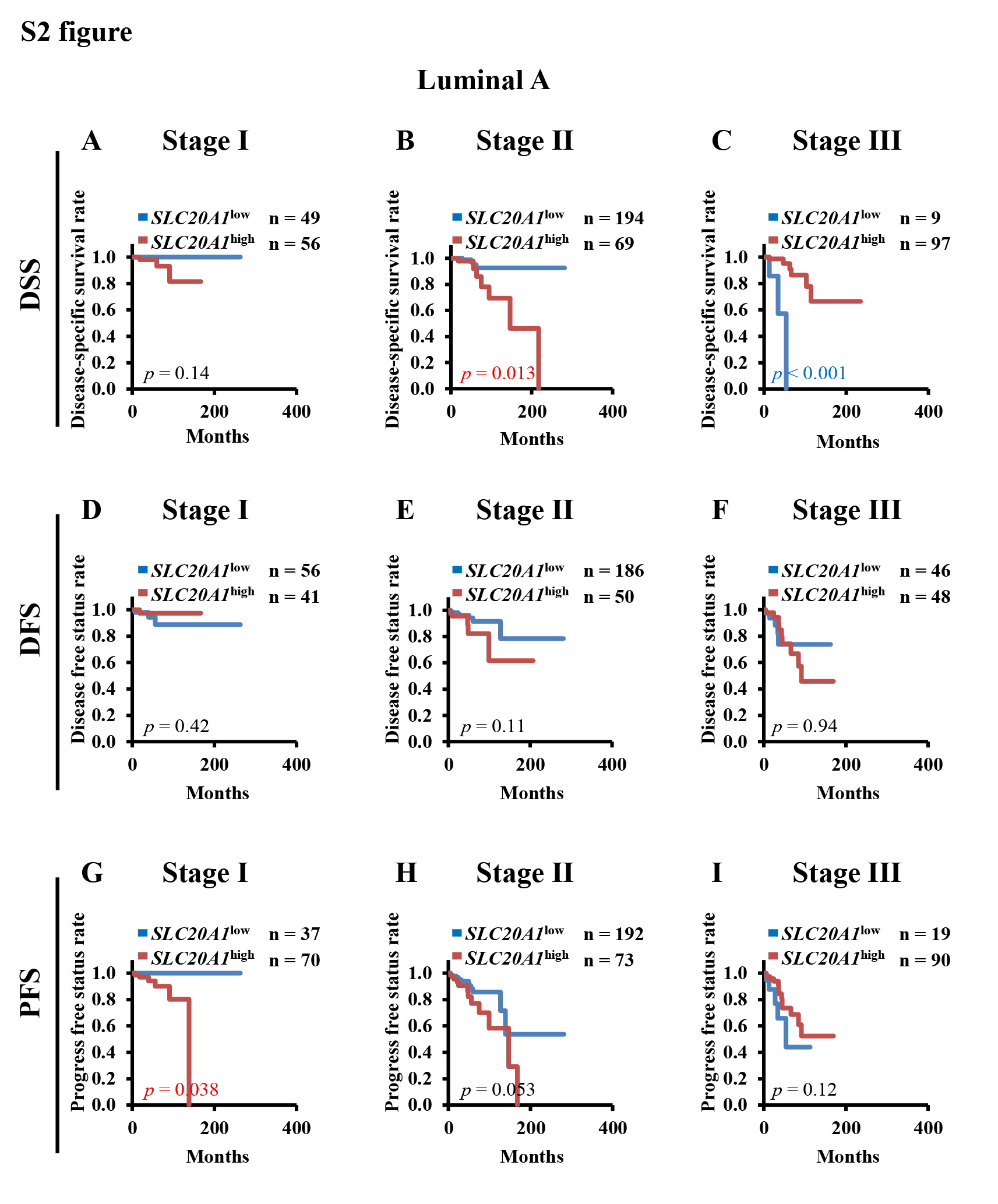

Supplement: S2 Fig — Kaplan-Meier analyses of patients with luminal A breast cancer with a high solute carrier family 20 member 1 (SLC20A1) expression (SLC20A1high) and a low SLC20A1 expression (SLC20A1low) at each stage. (A-F) Kaplan-Meier analyses comparing disease-specific survival (DSS), disease-free status (DFS) and progression-free status (PFS) in patients with luminal A breast cancer between the SLC20A1high and SLC20A1low groups. (A) DSS in tumor stage I. (B) DSS in tumor stage II. (C) DSS in tumor stage III. (D) DFS in tumor stage I. (E) DFS in tumor stage II. (F) DFS in tumor stage III. (G) PFS in tumor stage I. (H) PFS in tumor stage II. (I) PFS in tumor stage III. (TIF) [file pone.0268799.s002.tif]

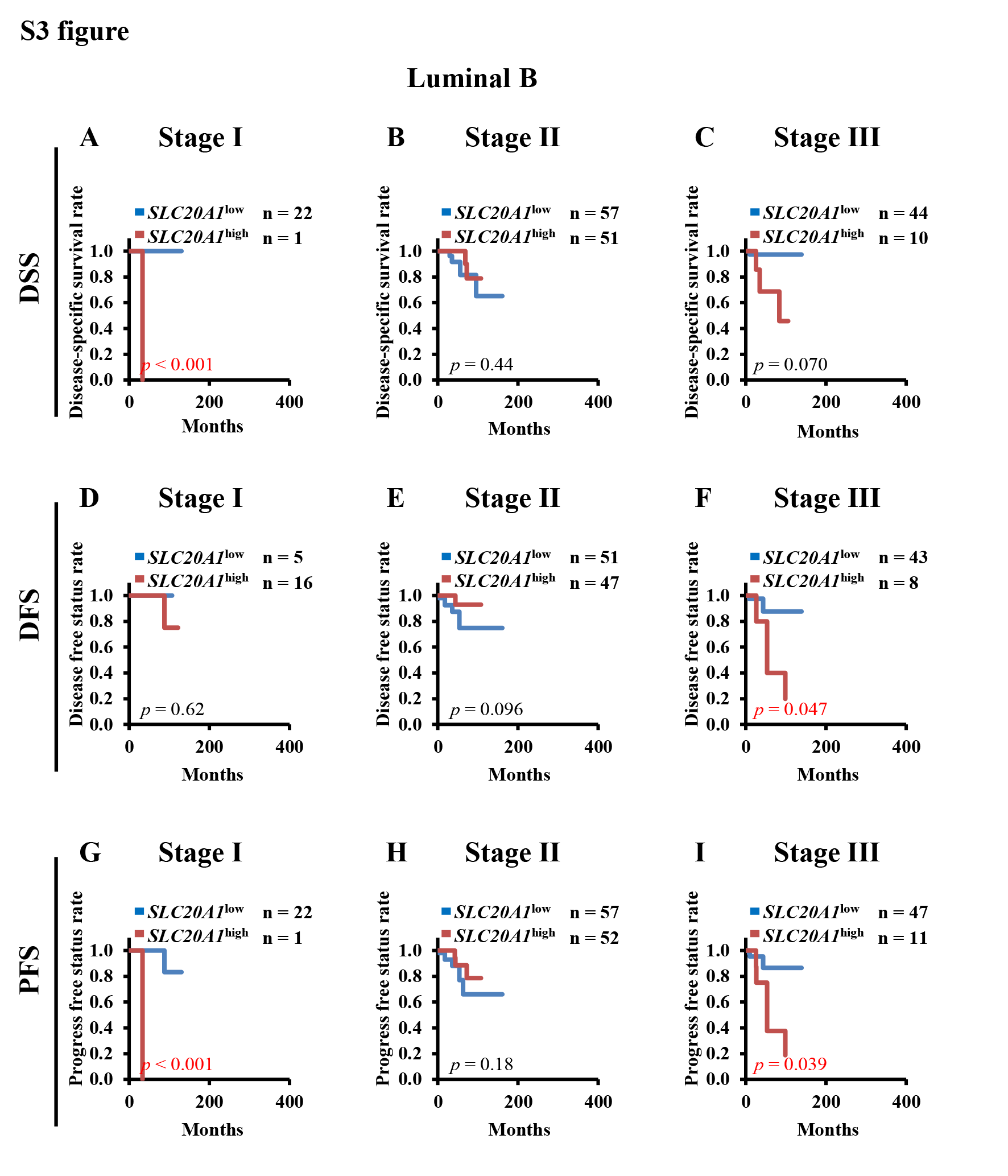

Supplement: S3 Fig — Kaplan-Meier analyses of patients with luminal B breast cancer with a high solute carrier family 20 member 1 (SLC20A1) expression (SLC20A1high) and a low SLC20A1 expression (SLC20A1low) at each stage. (A-F) Kaplan-Meier analyses comparing disease-specific survival (DSS), disease-free status (DFS) and progression-free status (PFS) in patients with luminal B breast cancer between the SLC20A1high and SLC20A1low groups. (A) DSS in tumor stage I. (B) DSS in tumor stage II. (C) DSS in tumor stage III. (D) DFS in tumor stage I. (E) DFS in tumor stage II. (F) DFS in tumor stage III. (G) PFS in tumor stage I. (H) PFS in tumor stage II. (I) PFS in tumor stage III. (TIF) [file pone.0268799.s003.tif]

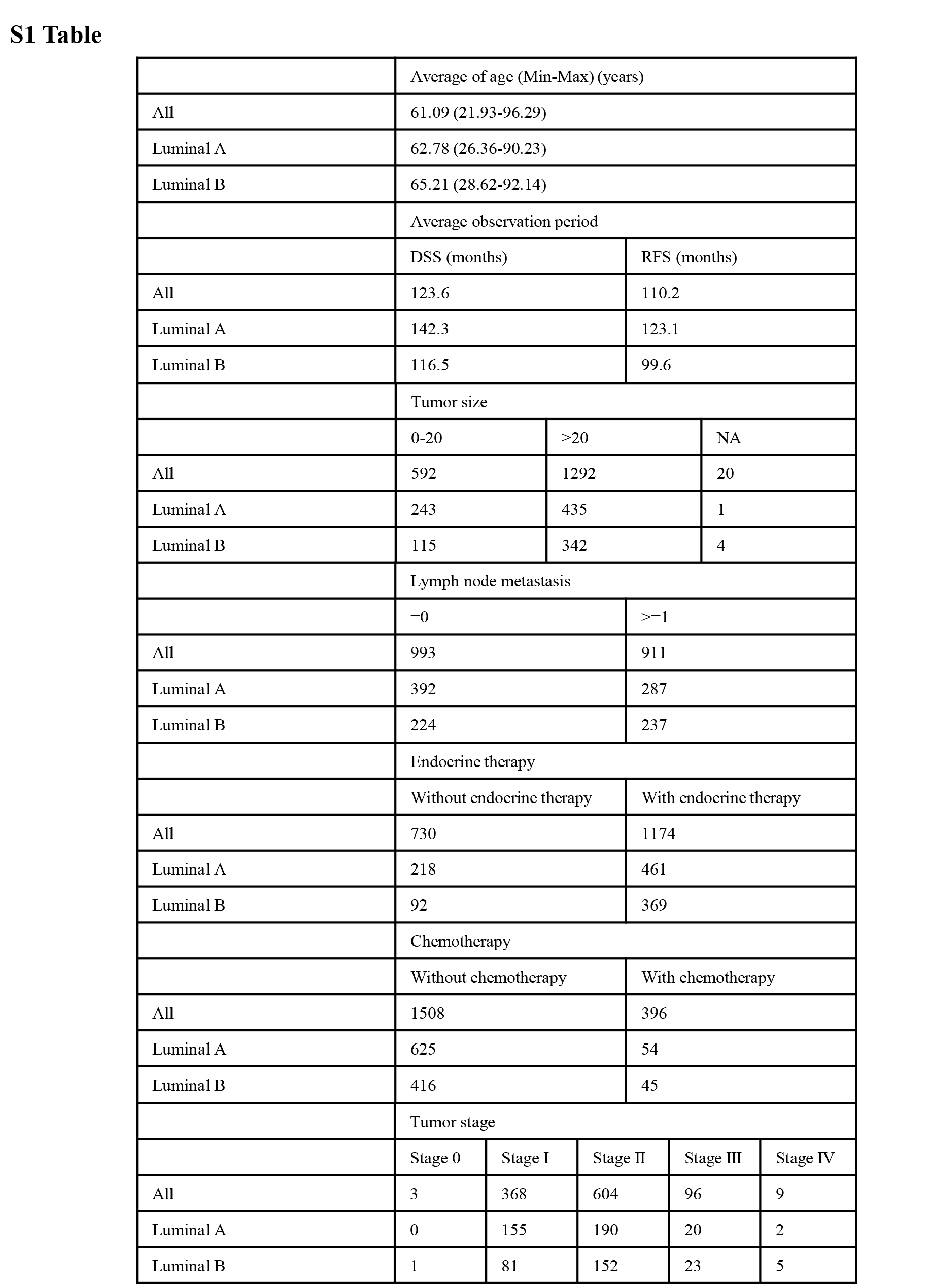

Supplement: S1 Table — (TIF) [file pone.0268799.s004.tif]

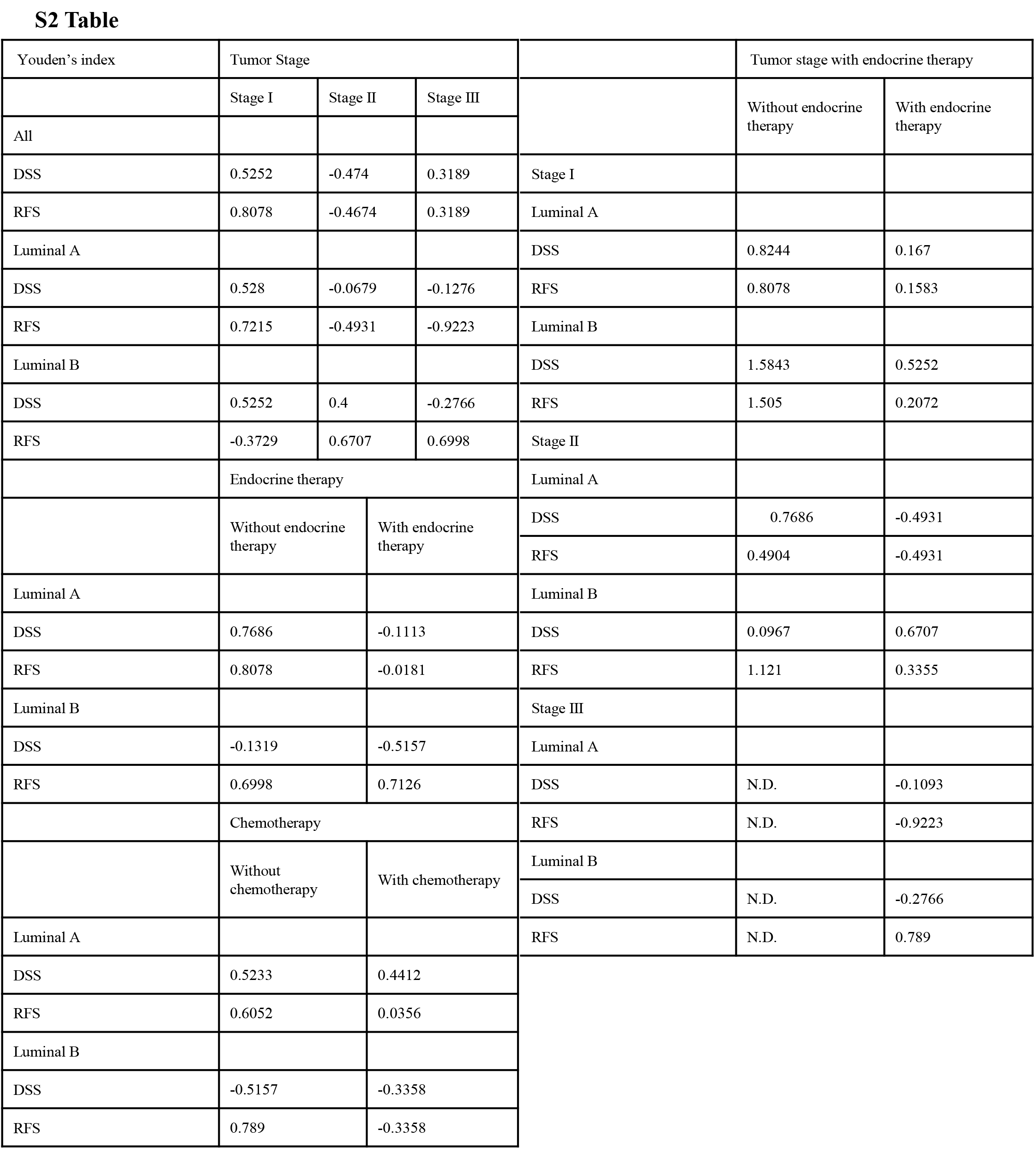

Supplement: S2 Table — (TIF) [file pone.0268799.s005.tif]

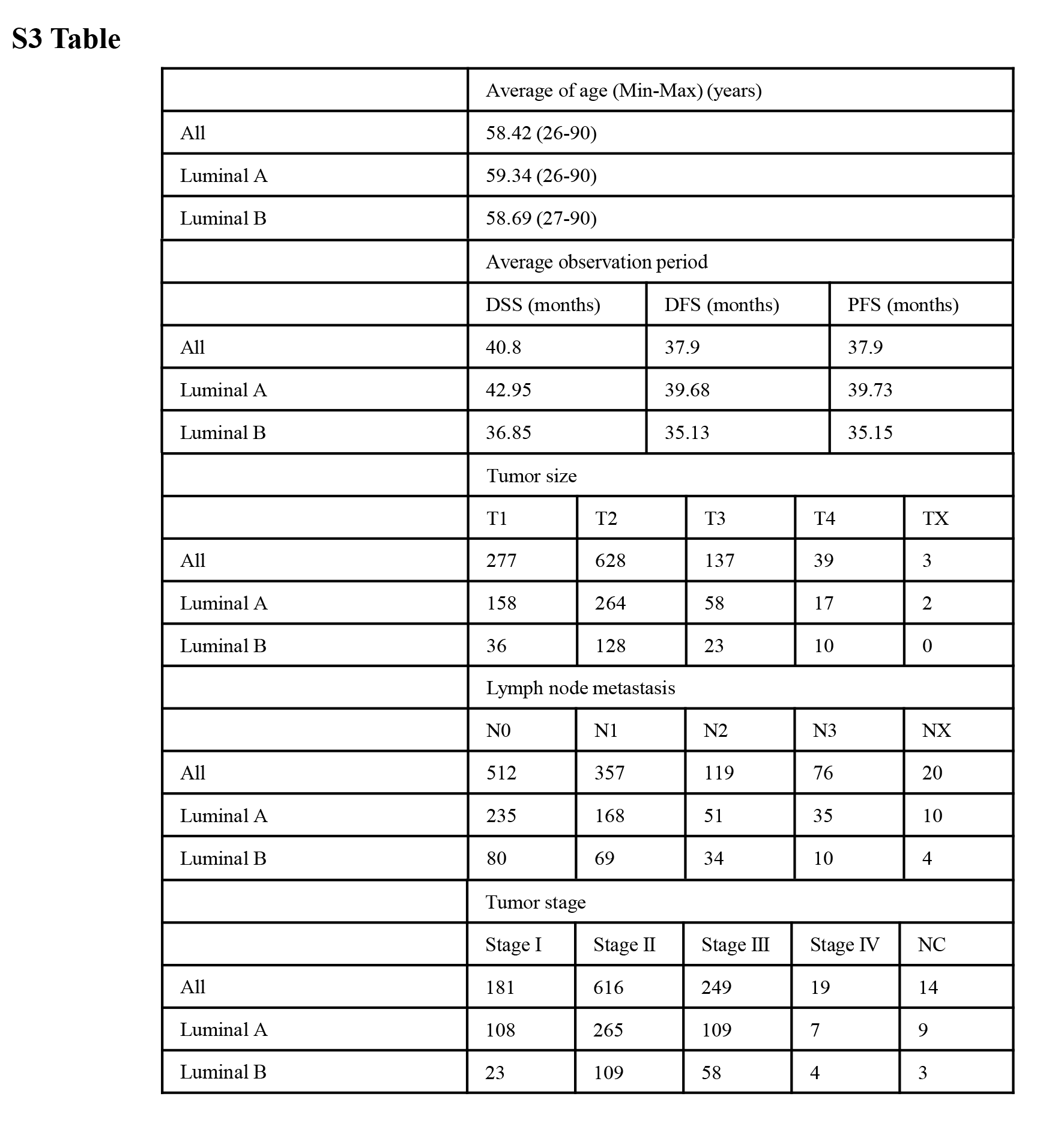

Supplement: S3 Table — (TIF) [file pone.0268799.s006.tif]

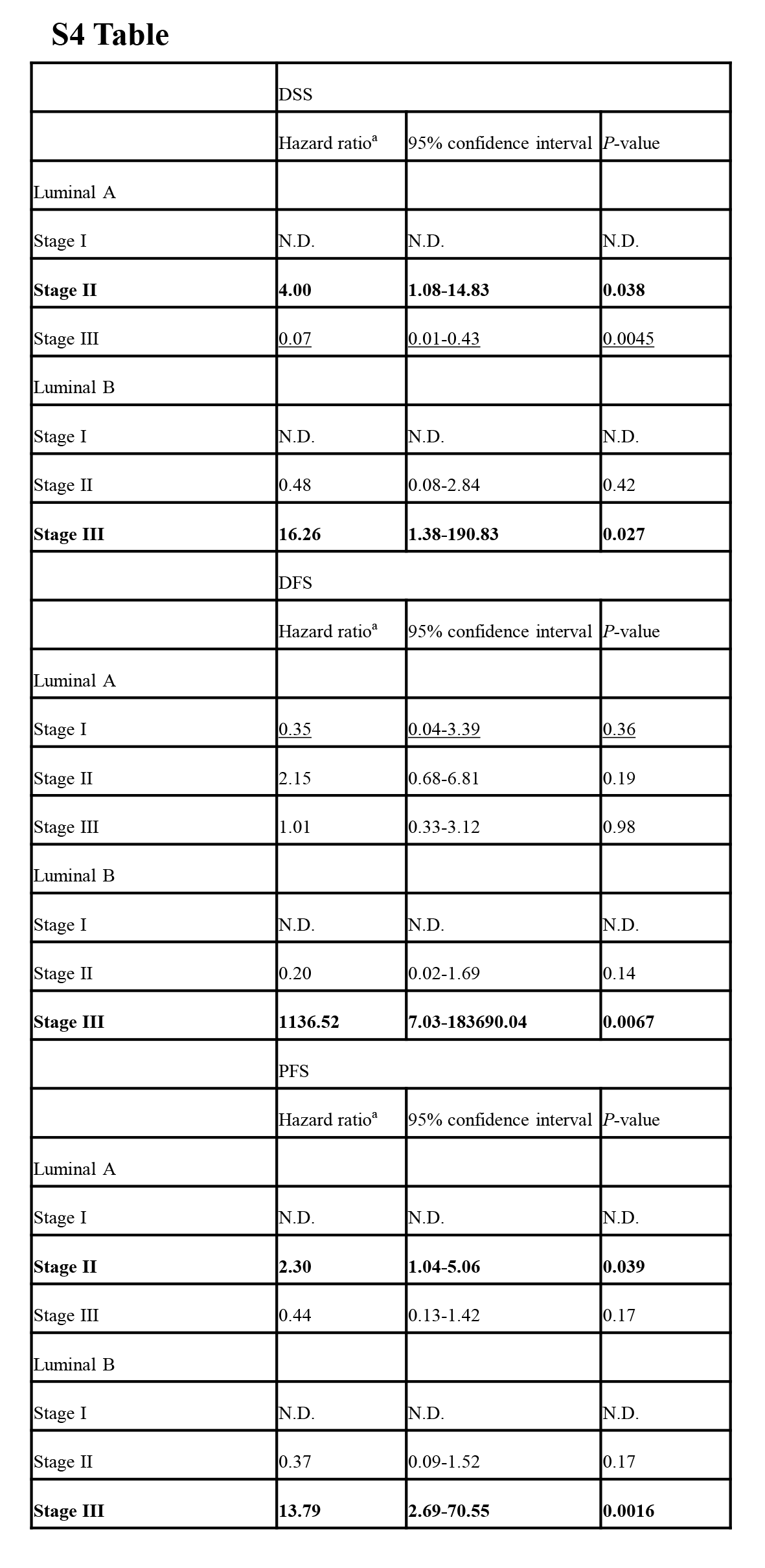

Supplement: S4 Table — (TIF) [file pone.0268799.s007.tif]

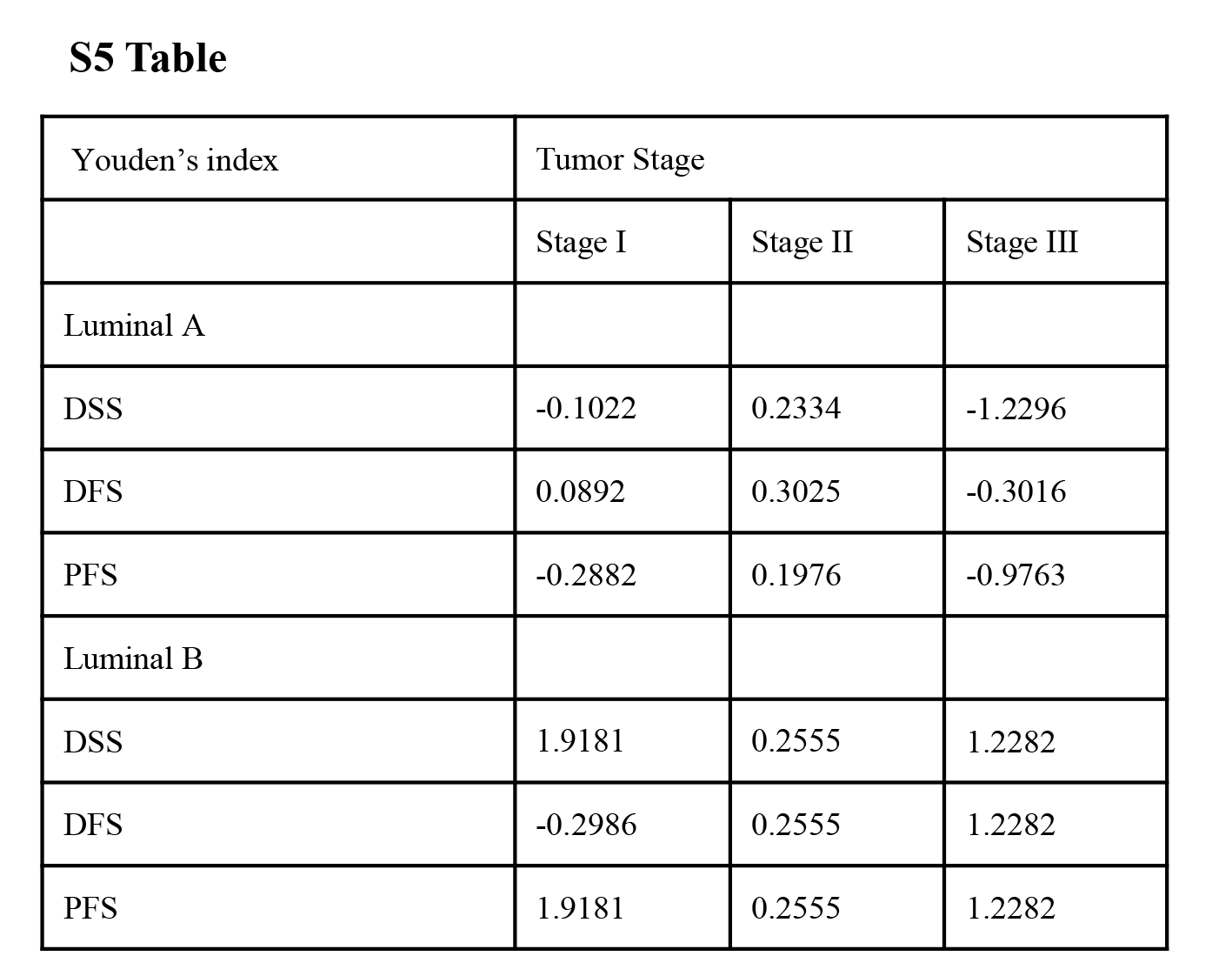

Supplement: S5 Table — (TIF) [file pone.0268799.s008.tif]
