# Supplementary material for: High expression of SLC20A1 is less effective for endocrine therapy and predicts late recurrence in ER-positive breast cancer
Source: PLoS One. 2022 May 23;17(5):e0268799. doi: 10.1371/journal.pone.0268799 (PMC9126382; doi:10.1371/journal.pone.0268799)
Supplement: S1 Text — (DOCX) [file pone.0268799.s009.docx]

**TCGA PanCancer Atlas dataset analyses**

**TCGA PanCancer Atlas dataset**

TCGA PanCancer Atlas dataset was downloaded from the cBioportal (http://cbioportal.org) on February 25, 2022. The clinicopathological data from these patients are summarized Table SIII. The average values of the ages at the time of diagnosis in the entire cohort and in patients with Luminal A and Luminal B disease are as follows [all, 58.42 years (range, 26-90 years); luminal A, 59.34 years (range, 26-90 years), luminal B, 58.69 years (range, 27-90 years)]. This TCGA PanCancer Atlas dataset contains mRNA expression profile data (n=1,082). The optimal cut-off thresholds were defined using Youden’s index to assign the patients into the *SLC20A1*^high^ and low-expression (*SLC20A1*^low^) groups through receiver operating characteristic (ROC) analysis. ROC analysis was performed using SLC20A1 expression and disease-specific survival (DSS) or disease-free status (DFS) or progression-free status (PFS) for each group divided, and Youden’s index was calculated (S5 Table).

**Analysis of patient prognosis using the Kaplan-Meier method**

Survival curves based on DSS, DFS and PFS were plotted using the Kaplan-Meier method. The curves were compared between the *SLC20A1*^high^ and *SLC20A1*^low^ groups using the log-rank (Cochran-Mantel-Haenszel) test. Kaplan-Meier survival curves were produced using BellCurve for Excel version 3.00 (Social Survey Research Information Co., Ltd.).

**Analysis of patient prognosis using the multivariate Cox regression method**

Multivariate Cox regression analysis was performed to evaluate the influence of a high and low *SLC20A1* gene expression on patient outcomes and to estimate the adjusted hazard ratios (HRs) for the *SLC20A1*^high^ group compared with the *SLC20A1*^low^ group as regards DSS, DFS or PFS. The ages at diagnosis were adjusted as a confounding factor to exclude the effect of age. The level of significance was set at 5% (two-sided). Multivariate Cox regression analyses were performed using BellCurve for Excel version 3.00 (Social Survey Research Information Co., Ltd.).
